# Large-Scale Parameter Estimation for Crystal Structure Prediction. Part 1: Dataset, Methodology, and Implementation

**DOI:** 10.1021/acs.jctc.4c01091

**Published:** 2024-11-12

**Authors:** D. H. Bowskill, B. I. Tan, A. Keates, I. J. Sugden, C. S. Adjiman, C. C. Pantelides

**Affiliations:** † Department of Chemical Engineering, Sargent Centre for Process Systems Engineering and Institute for Molecular Science and Engineering, Imperial College London, London SW7 2AZ, U.K.; ‡ Process Studies Group, Syngenta, Jealott’s Hill International Research Centre, Bracknell, Berkshire RG42 6EY, U.K.

## Abstract

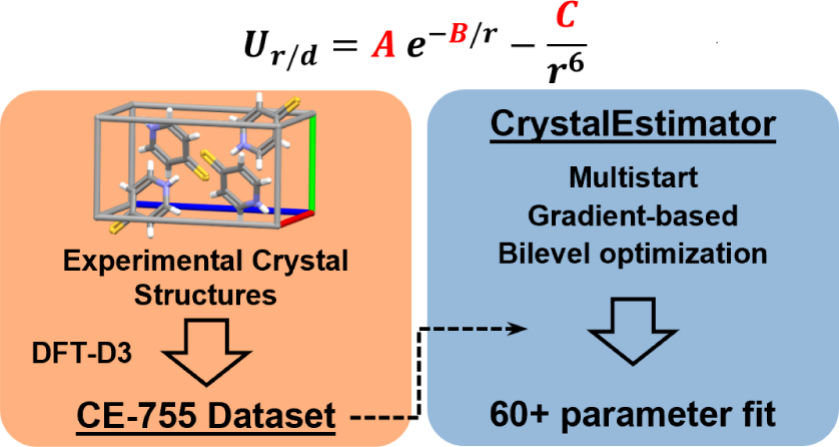

Crystal structure prediction (CSP) seeks to identify all thermodynamically accessible solid forms of a given compound and, crucially, to establish the relative thermodynamic stability between different polymorphs. The conventional hierarchical CSP workflow suggests that no single energy model can fulfill the needs of all stages in the workflow, and energy models across a spectrum of fidelities and computational costs are required. Hybrid *ab initio*/empirical force-field (HAIEFF) models have demonstrated a good balance of these two factors, but the force-field component presents a major bottleneck for model accuracy. Existing parameter estimation tools for fitting this empirical component are inefficient and have severe limitations on the manageable problem size. This, combined with a lack of reliable reference data for parameter fitting, has resulted in development in the force-field component of HAIEFF models having mostly stagnated. In this work, we address these barriers to progress. First, we introduce a curated database of 755 organic crystal structures, obtained using high quality, solid-state DFT-D calculations, which provide a complete set of geometry and energy data. Comparisons to various theoretical and experimental data sources indicate that this database provides suitable diversity for parameter fitting. In tandem, we also put forward a new parameter estimation algorithm implemented as the CrystalEstimator program. Our tests demonstrate that CrystalEstimator is capable of efficiently handling large-scale parameter estimation problems, simultaneously fitting as many as 62 model parameters based on data from 445 structures. This problem size far exceeds any previously reported works related to CSP force-field parametrization. These developments form a strong foundation for all future work involving parameter estimation of transferable or tailor-made force-fields for HAIEFF models. This ultimately opens the way for significant improvements in the accuracy achieved by the HAIEFF models.

## 1 Introduction

Organic crystalline solids are a key delivery mechanism in product formulations for a number of industries, such as pharmaceuticals and agrochemicals. The physical properties and the thermodynamic stability of the solid phases of organic compounds are dependent on 1) the chemical compound in question, and 2) the arrangement and conformations of all molecules in a 3-dimensional lattice. Polymorphism, the possibility of a compound adopting multiple crystalline forms in the solid state, plays a key role in product development, such as during solid form selection. Different polymorphs can be observed experimentally, either under different conditions or concomitantly, and the differences in their structure can have a profound impact on the physical properties of the crystal. The effects of polymorphism are widespread, hence any early knowledge pertaining to the thermodynamic stability of crystal structures under a given set of conditions can be highly valuable in accelerating product development and avoiding disastrous situations during manufacturing.^1,2^ Unfortunately, there are limited guidelines for knowing which crystallization conditions might promote polymorph formation, or if a polymorph even exists.^3^ As such, it is not possible to be certain that an experimental screen has uncovered all polymorphs, and gaining increased confidence requires a significant investment in cost and time.

To complement experimental screens, crystal structure prediction (CSP) methods have been the subject of extensive research. The aim of CSP is to predict all polymorphs of a given compound *in silico*, with an emphasis on the identification of the most energetically stable structures, given as little information as the molecular connectivity of the molecule.^4^ Over the last 25 years, CSP has gradually progressed into a practical tool, which has been successfully demonstrated on a growing number of compounds when employing a hierarchical workflow that combines multiple models across several stages.^5^ Many differing models of crystal energy have been proposed for use within this workflow,^6^ but almost all incorporate some form of force-field. Among other differences, the force-field parametrization is an important factor that distinguishes these models and influences their accuracy. Data used in obtaining force-field parameters may be system-specific^7^ or involve a range of systems.^8^ These data could be based on the analysis of entire crystals^9^ or of molecular-clusters^10^ and could be derived from computations^10,11^ or, in the case of whole-crystal data, it could come from experimental measurements.^8,9^ Given the importance of the force-field parameters in achieving meaningful results^12^ and the often incremental manner in which such parameters have been derived, it is valuable to investigate whether more appropriate parameters could be used. In the following sections, we begin by examining some of these models and the associated parametrization strategies, identifying limitations in the approaches used to date before proposing a new method to parametrize the force-field for a widely used CSP model.

### 1.1 Lattice Energy Models in CSP

At a given temperature, pressure, and composition, stable and metastable polymorphs of one or more organic compounds are usually postulated to correspond to low-lying minima on the Gibbs free energy surface. However, evaluating the Gibbs free energy accurately within the extensive breadth of a CSP search is highly challenging, and in most cases computationally impractical. In practice, several approximations are applied. In particular, thermal contributions at low temperatures and the effects of pressure under atmospheric conditions are neglected initially. In effect, the Gibbs free energy is instead approximated by the lattice energy of the system at 0 K and 0 Pa. An important requirement in many CSP methodologies is thus to develop models of lattice energy that are sufficiently accurate to determine relative lattice energy differences between polymorphs, while remaining computationally efficient enough to allow a complete exploration of the landscape of polymorph candidates.

Due to practical limits on computational resources, solid-state density functional theory with dispersion corrections (DFT-D) is the most accurate modeling framework that is routinely applicable in the context of CSP methodologies. In many cases, DFT-D has been shown to reliably predict polymorph energy differences,^13−15^ despite well-known issues in some cases with conformational polymorphism^16,17^ and delocalization errors.^18^ Even so, the precision of DFT-D calculations still comes at a significant computational cost which often makes it prohibitive to apply DFT-D to more than a small number of structures in the CSP search. As such, the use of DFT-D is often restricted to providing a final ranking of polymorphs in the later stages of a hierarchical CSP study, or to perform *a posteriori* free energy calculations.^13^ In the earlier stages of the CSP workflow, force-field models are usually used due to their comparatively low computational cost. Transferable force-field models typically used in molecular dynamics applications were common in the pioneering days of CSP,^19,20^ but models that incorporate some form of system-specific customization are increasingly being used.^5,6^ Tailor-made force-fields (TMFF) have been successfully applied in CSP methodologies and resemble a typical molecular dynamics force-field, but the model must be reparameterized from solid-state DFT-D calculations for each system to obtain a customized empirical model of molecular interactions.^21^ Work has also been carried out on the development of bespoke potentials using SAPT(DFT) where all terms in the classical model are derived from *ab initio* calculations on the system of interest.^10,22−24^ For instance, in SAPT(DFT) derived models, parameters are either tailored directly to the potential energy surface of a molecule from theoretical calculations on hundreds or thousands of dimer interactions,^22,23^ or are determined *ab initio* from the properties of the molecular charge density.^10,24^ More recently, machine-learning based methods have gained increased interest within CSP for molecular systems. However, limitations on computational costs and data availability hinder the viability of purely machine-learned models. Instead, machine-learning models have been implemented as corrections over non-machine learning CSP methods, reducing the volume of training data required to a manageable level.^25,26^ While the efficacy of these methods have been demonstrated in relatively small and rigid molecules, their effectiveness on more flexible molecules has yet to be reported.

Another class of models, which we henceforth refer to as hybrid *ab initio*/empirical force-field (HAIEFF) models, has been well-established within common CSP methodologies.^27^ In their most basic form, HAIEFF models partition the crystal lattice energy (*U*_*latt*_) into independent energetic contributions that include the cost of deforming the molecules in the crystal from their gas phase conformation (Δ*U*_*intra*_), intermolecular electrostatic interactions (*U*_*elec*_), and finally a residual term (*U*_*res*_):
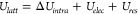
1The first two terms are typically described by tailored surrogate models constructed from *ab initio* calculations carried out on an isolated molecule, while the remaining residual interactions in the crystal (*U*_*res*_) are modeled using a transferable, empirical force-field. These residual interactions include repulsive/dispersive effects from Pauli exclusion and electron correlation respectively, inductive effects, many-body effects, as well as any systematic errors in the *ab initio* terms.^7^ Because the effects of Pauli exclusion and electron correlation tend to dominate the other residual terms, the residual interactions are often just referred to as the repulsion/dispersion interaction. To most effectively capture the combined effects of these various interactions, the force-field should be parametrized to suitable crystal data. The modular construction of HAIEFF models offers flexibility in handling the trade-off between accuracy and computational cost, which facilitates their use at multiple stages of a CSP investigation. For example, simple point-charge based electrostatics with limited molecular flexibility are initially used in the candidate generation stage, followed by a more detailed multipole description of the electrostatics and extensive molecular flexibility in subsequent refinement stages.^6^ This type of model and workflow were employed by several groups in the sixth blind test with good success in the identification of (meta)stable polymorphs.^5^

Although HAIEFF models are unlikely to reach the level of accuracy necessary to supplant DFT-D as a final ranking approach, a terminal DFT-D calculation is meaningless if the energy models which precede it cannot deliver the correct structures for ranking. This is the case if a cheaper energy model fails to identify valid polymorphs as energy minima or if the identified minima are beyond the energetic cutoff of structures carried forward to the next stage of the hierarchical study. For this reason, continued improvement of HAIEFF model accuracy is still a valuable endeavor. A leading cause for inadequate HAIEFF model accuracy may be poor parametrization of the force-field component, for instance if the reference data used is heavily biased toward crystal geometry over crystal energy. Moreover, recognizing the evolving role of HAIEFF models within the larger CSP workflow, and in the spirit of “blind” CSP where experimental data is absent, reference data representative of the DFT-D landscape may be a more sensible yardstick for accuracy. Another contributor to the inaccuracy of HAIEFF models is the (mis)use of repulsive/dispersive parameters in a manner that is inconsistent with their derivation. For example, during a CSP search, use of *ad hoc* repulsive/dispersive parameters is common, even if the parameters were originally derived using electrostatic and/or intramolecular models different from the models employed in the ongoing CSP search. There is therefore a clear drive for the development of tools to reliably and efficiently obtain transferable parameter sets for this class of models.

### 1.2 Parameter Estimation in CSP

Parameter estimation is a critical task in the development of force-field models, and the quality of the parameters used in the force-field are integral to model performance. Within the formulation of HAIEFF models, atom types are assigned to each site in a molecule. A pair potential is used to describe the interaction between each possible atom type-atom type pairing. By keeping a consistent definition of the atom types, the parameters describing each pairwise interaction can remain unaltered between different molecules, hence the “transferable” attribute of the repulsion/dispersion term in HAIEFF force-fields. Transferable potentials help provide practical limits on the number of parameters describing the force-field, although they can often lack an appropriate consideration of the effects of the local molecular environment on the repulsive/dispersive interactions. Within this general framework, the FIT^8,28−32^ and Williams^9^ parametrizations emerged as early forebearers of HAIEFF force-fields.

The FIT parametrization^8,28−32^ distinguishes atom types based solely on the corresponding chemical element, with the exception of hydrogen atoms which were allowed to adopt different parameter values if bonded to a polarizing element such as oxygen or nitrogen. Crucially, only homoatomic interaction parameters were explicitly parametrized while cross-interactions between heteroatomic atom types were calculated using predetermined combining rules. For a set of reference crystal structures, parameters were then optimized so as to minimize the forces exerted on each atom at the experimental crystal geometry, as well as the difference between computed lattice energies and measured sublimation energies. A flaw of the empirical force-fields derived using this approach is that when they are used within an energy minimization, the resulting crystal geometry may no longer be a close match with the reference geometry. The parametrization proposed by Williams^9^ was derived using a similar methodology, but atom types were further distinguished to account for bonded-neighbors and the local molecular environment of each site. This more elaborate atom typing scheme was applied to both heavy atoms in addition to hydrogen atoms.

It is worth noting that both the FIT and Williams potentials (at least as described in ref (8) and ref (33), respectively) characterized electrostatic interactions using multipoles derived from isolated-molecule quantum mechanical (QM) calculations. Therefore, strictly speaking, they should not be used in conjunction with other electrostatic descriptions (e.g., point-charges). Moreover, while the use of combining rules is convenient to manage the number of model parameters, there is no rigorous theoretical justification of their validity. More recent work has highlighted the importance of determining cross-interaction parameters explicitly, as well as providing parametrizations consistent with more modern electrostatic models.^33,34^ These parametrizations have shown notable improvements for both energetic and geometric predictions of experimental crystal structure. Finally, in these modern methods, parametrization has also shifted toward minimizing geometric and energetic discrepancies of the *relaxed* crystal geometries relative to the reference structures. While this significantly increases the complexity of the parameter estimation procedure, it is theoretically more robust than the earlier approaches. Since any experimentally observed reference structures must also be a local minimum on the lattice energy landscape, matching geometry and energy specifically at the relaxed crystal structure strictly enforces this local optimality criterion. In contrast, the FIT and Williams parametrizations only loosely compel optimality by minimizing forces at the experimental geometry.

Another key consideration is the reference data used to fit parameters, where it has been commonplace to use energetic and geometric data from experimental sources.^33,34^ Geometric data for a wide variety of structures are readily available from the Crystal Structure Database (CSD) which recently surpassed one million entries.^35^ The geometries of these crystal structures are typically determined from experimental methods such as neutron diffraction, or more commonly, single crystal or powder X-ray Diffraction (XRD). With the exception of hydrogen atoms resolved by XRD, the uncertainties in experimentally determined geometries are expected to be small. When used directly within CSP force-field parametrization, some error might propagate from neglecting the thermal expansion of the crystal at finite temperatures. Fortunately, these effects are generally considered to be quite minor. Energetic data are commonly derived from sublimation enthalpy experiments. Sublimation enthalpies are usually obtained at room or elevated temperatures and contributions from vibrational effects can be quite significant. As such, there are a number of issues with the use of sublimation enthalpy data within this context. First, the results of sublimation enthalpy experiments lack consistency. The standard deviation of reported sublimation enthalpies across experiments is estimated in the region of ±4.9 kJ/mol,^36^ and in some cases uncertainties have been estimated to be as large as ±10 kJ/mol.^37^ This may be as a result of impurities in the samples, or differences in the experimental methods used. Second, a simple thermal correction is often used to negate vibrational effects.^34,38^ By comparison to DFT-D lattice dynamics calculations,^37^ typical errors in the thermal correction are over 2 kJ/mol on average, and sometimes as large as 6 kJ/mol. As such, the precise determination of reference lattice energies from sublimation enthalpies is marred by much uncertainty. This problem is further compounded by the fact that trustworthy sublimation enthalpy results are often in short supply. This scarcity of data is particularly apparent for organic compounds containing a mixture of “less common” elements beyond carbon, hydrogen, nitrogen, and oxygen. That being said, even among the “more common” elements, curation of sufficiently large and reliable training sets is challenging and studies are often limited to including only a few energetic data points in the training set.^9,34,38^

An alternative to using experimental data is to derive energetic and geometric data using synthetic data derived from more accurate, but computationally expensive, models. In recent years, much work has been carried out on the development of post-Hartree–Fock fragmentation approaches^39,40^ and fixed-node diffusive quantum Monte Carlo^41^ for the study of organic molecular crystals, but such methods are not easily applied in routine applications and their computational cost can be prohibitive. DFT-D perhaps still offers the best compromise between the accuracy and the cost of predictions, and is widely available in commercial and academic DFT-D packages such as VASP^42^ or CRYSTAL.^43^ The performance of DFT-D in the prediction of the lattice energies of organic solids has been assessed in a number of benchmark studies. The work of Otero-de-la-Roza and Johnson^44^ and Reilly and Tkatchenko^37^ led to the construction of the X23 test set, a set of 23 organic crystal structures with reference lattice energies derived from a combination of reliable sublimation enthalpy results and accurate vibrational corrections from lattice dynamics. Reilly and Tkatchenko^37^ found that the use of the PBE0 hybrid-functional^45^ and the MBD dispersion correction^46^ yields a mean absolute deviation of 3.92 kJ/mol compared to the experimental data. Brandenburg and Grimme^47^ found that the PBE functional^48^ with the D3 dispersion correction^49^ achieved a mean absolute deviation of 4.48 kJ/mol. Comparing the performance of an array of functionals, Moellmann and Grimme^50^ and Brandenburg et al.^51^ showed that the TPSS functional^52^ and PBE functional both perform well when paired with the D3 dispersion correction, with mean absolute deviations of 3.77 and 4.60 kJ/mol, respectively. It is of note that these errors are within the uncertainty of sublimation enthalpy experiments making estimates of the true accuracy of these methods challenging. Further benchmarking of DFT-D in CSP applications was carried out using the POLY59 benchmark set^14^ and demonstrated that the TPSS and PBE functionals with the D3 correction are sufficiently accurate to identify the most stable polymorphs of all molecules used in the sixth blind test.^5^ The reported accuracy of these calculations adds credence to the use of theoretical results in lieu of reliable experimental data.

The use of synthetic data sources for CSP model development is not novel. CSP models such as TMFFs^21^ and other recent approaches,^53^ contain customized model parameters derived from solid-state DFT calculations for the target compound under study as an integral step of the CSP workflow. The use of synthetic data is also implicit in the derivation of tailored SAPT(DFT) potentials since they are parametrized from data on hypothetical monomers and dimers. Machine-learning models, by nature, require more data to train than could ever be provided by experimental sources, hence synthetic data are always used.^25,26^ Overall, investigations using synthetic reference data have predominantly centered on developing customized, system-specific force-field models, while little research has been performed on similar use of synthetic data in the development of potentials for HAIEFF-type models. In this context, some attempts to fit the empirical component to synthetic data sources include using energies calculated from the interaction of dimers,^54^ and more recently in the customization of the empirical potential to solid-state DFT-D calculations on a specific system.^7^

The slow progress toward extending this approach to a *transferable* HAIEFF force-field may stem from two reasons. First, to our knowledge there does not currently exist an appropriate, open-source database of synthetic data suitable for force-field parametrization. The computational cost of generating a suitable database is considerable given that transferability requires such a dataset to cover a wide variety of molecular systems without compromising on the accuracy of the theoretical calculations. In this context, HAIEFF models benefit from the fact the empirical force-field attempts to describe only the residual interactions and thus requires fewer fitting parameters. The other parameters describing the intramolecular and electrostatic energy are derived independently from *ab initio* calculations on the system of interest. This means that the quantity of reference data required to derive reliable parameter estimates is significantly reduced. In contrast, a TMFF that attempts to parametrize empirical descriptors for all interactions in the crystal would require a more extensive dataset. As an example, when a TMFF was parametrized for a relatively small molecule like cyclohexane-1,4-dione (16 atoms), a combination of 157 crystal and conformational data points were employed.^21^ 89 of these data points were used for fitting the intramolecular and electrostatic interactions that would have otherwise been excluded from the empirical parts of the HAIEFF model. Moreover, transferable HAIEFF force-fields might be more data-hungry than their molecule-specific counterparts, but once generated the reference data can be reused in all future transferable HAIEFF force-field parametrizations making it an investment that is “repaid” over time. Conversely, in molecule-specific models, each set of synthetic reference data generated is a sunk cost for that particular system.

A secondary barrier to using synthetic data for transferable HAIEFF force-field parametrization is the absence of an appropriate tool for this purpose. Despite some of the parametrization attempts listed above, recent parameter estimation methodologies developed for HAIEFF models in the literature are computationally costly and limit the number of structures or parameters that can be optimized simultaneously.^7,33,34^ This means that a sequential optimization scheme must be used, where each parameter is optimized individually or as part of a small set, while the other parameters are fixed. Parameters determined from this sequential approach would be suboptimal compared to if all parameters could be fit together.

In this study, we tackle these two barriers against the parametrization of HAIEFF force-fields using synthetic data. First, we curate a database of 755 crystal structures, with corresponding optimal lattice energies and geometries derived from high-quality, solid-state DFT-D calculations for each crystal. Each entry in this database, while initially very expensive to generate, can be reused multiple times in the fitting of empirical force-fields that are consistent with different electrostatic and intramolecular energy models. The use of high-fidelity DFT-D reference data not only ensures that we are fitting to reliable data but is also congruent with the overarching CSP workflow wherein DFT-D results are usually the “final solution” that is accepted. Second, we address the shortcomings in many current approaches to parameter estimation of HAIEFF models and present an efficient algorithm for multivariate optimization of model parameters. Our approach relies on gradient-based optimization techniques to rapidly solve large-scale parameter estimation problems. To do this, we establish a novel route for determining analytical derivatives of the parameter estimation merit function. The determination of the crystal structure for a given set of empirical parameter values is performed through our newly developed software library for performing lattice minimization of rigid molecules known as Crystal Structure Optimizer—Rigid Molecules (CSO-RM).^11^ CSO-RM supports calculations for either point-charge or multipole based electrostatic models, and also computes any additional analytical derivatives required by our parameter estimation algorithm. The combined implementation of CSO-RM and the parameter estimation algorithm is known as CrystalEstimator. In this paper we present our parameter estimation algorithm as implemented in CrystalEstimator and the development of the reference structure database from DFT-D calculations. The effectiveness of the implementation is evaluated on a series of test problems of increasing difficulty to demonstrate the benefits of our approach. Application of the code in the development of transferable parameter estimates will be reported in part 2 of this paper series.

Our paper is organized as follows. In Section 2 we introduce an efficient and reliable methodology for estimation of potential parameters consistent with HAIEFF models. In Section 3 we present the implementation of the code CrystalEstimator. In Section 4 we present the construction of the DFT reference datasets, as well as the settings and test problems used in our investigation. In Section 5, we investigate the performance of the proposed algorithm, focusing on its speed and robustness. Finally, concluding remarks are made in Section 6.

## 2 Methodology

### 2.1 Energy Model Formulation

The general mathematical formulation of hybrid *ab initio*/empirical force-field models in CSP is based on a strict separation of intramolecular and intermolecular contributions to the lattice energy (*U*_*latt*_):
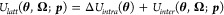
2Δ*U*_*intra*_ is the intramolecular energy penalty for deforming each molecule in the asymmetric unit cell from its unconstrained *in vacuo* conformation to its conformation in the crystal, while *U*_*inter*_ is the intermolecular energy contribution to lattice energy. **θ** denotes the vector of molecular conformational variables (e.g., bond lengths, bond angles, and torsion angles). **Ω** relates to all other geometric variables such as the centers of mass of all molecules in the asymmetric unit cell in fractional coordinates (), the variables defining molecule orientation (i.e., Euler angles) for each molecule in the asymmetric unit cell, and the lattice lengths and angles of the unit cell. The intermolecular energy model also depends on the empirical model parameters denoted ***p***. For clarity in the rest of this paper, we use a semicolon “;″ to make the distinction between variables (which come before the “;″) and constants (which come after the “;″), as shown in eq 2.

The lattice energy has additional implicit fixed dependencies not shown in eq 2. This includes the space group symmetry of the unit cell which relates the positions of symmetry related molecules in the unit cell to the asymmetric unit cell. The lattice energy is also dependent on other physics-based parameters in the model. These include electrostatic parameters,  (i.e., atom-centered multipoles or point-charges), which are dependent on the molecular conformation. Both the intramolecular and electrostatic parameters are supplied to the model in advance and are derived from isolated-molecule QM calculations.

The intermolecular energy model is comprised of two terms, the electrostatic energy, *U*_*elec*_, and a repulsion/dispersion energy component, ,
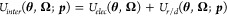
3The repulsion/dispersion component is inherently empirical and accounts predominantly for interactions arising from Pauli-exclusion and electron correlation. Due to its empirical nature,  implicitly contains other residual binding forces such as polarization, charge transfer, or electrostatic penetration effects that might be present in the reference data, but are less straightforward to model directly and hence are not explicitly included elsewhere in the model. The repulsion/dispersion term is often the leading contribution to lattice stability when considering van der Waals bound structures, although the electrostatic contribution can dominate for systems containing hydrogen bonds or ionic species. In this paper, the electrostatic term is modeled through the interactions of atom-centered point-charges or distributed multipoles. To mitigate issues related to conditional convergence,^55,56^ an Ewald summation is employed to evaluate the electrostatic term for all multipole ranks.

For the calculation of the repulsive/dispersive energy component, a pairwise potential function denoted  is used. This pairwise function describes the interaction between atom *i* in molecule *I* in the central unit cell, indexed **0**, with atom *j* in molecule *J* in a periodic image of the central unit cell, indexed ***l***. Several analytical forms can be used for this potential including the Lennard-Jones and Mie potentials, but we will focus on the Buckingham potential due to its near-ubiquitous use in modern CSP applications. The Buckingham potential between atoms  and ,
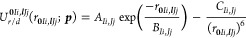
4is dependent on the Euclidean interatomic distance between the two atom sites, denoted . The interatomic distance can be determined from the variables defining the geometry of the crystal (i.e., ). At relatively close ranges, the exponential contribution to the Buckingham potential dominates and accounts for repulsion forces arising from the Pauli-exclusion principle. This decays rapidly with interatomic distance and gives way to first-order long-range London dispersive forces that decay with the interatomic distance to the negative sixth power. The model contains three parameters per atom–atom interaction (), influencing the shape and depth of the potential.

The repulsive/dispersive term can be evaluated by direct space summation of eq 4 over all atom sites and molecules in the central unit cell, **0**, and all periodic images of the central unit cell, ***l***, within a valid summation cutoff. The parameters used for the interaction between site  and  are typically determined from the atom type of each participating site. This ensures that species of the same type are treated identically and that the number of parameters does not become overwhelming. It has also been common in CSP to employ combining rules, whereby parameters describing the interactions between atoms of different types are estimated as a geometric or arithmetic average of their homoatomic atom–atom interactions. A geometric mean is often used for  and , whereas an arithmetic mean is usually chosen for the inverse of :
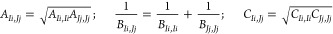
5

A well-known deficiency of the Buckingham potential appears at very close ranges. As  tends to zero, the exponential term converges to the value of  while the  term diverges asymptotically and dominates the former term. This leads to an unphysical attraction at very short ranges. In practice, this issue can be avoided as long as there is a sufficiently large energy barrier arising from the repulsive interaction at short-to-medium ranges. The size and location of this energy barrier is a function of parameters , , and  and so it is important that suitable values are chosen to effectively “insulate” against the unphysical parts of the Buckingham potential. However, during the parameter estimation process, if the values of several parameters related to the same pair potential are allowed to vary simultaneously, this can be challenging.

#### 2.1.1 Lattice Energy Minimization

The lattice energy of a (meta)stable crystal structure on the energy surface defined by the HAIEFF model is computed by local minimization of the energy function over the variables **Ω** and **θ**. With the inclusion of intramolecular energy contributions and molecular flexibility, changes in molecular conformation become a major factor in model complexity. This is not only due to the need to create detailed models for intramolecular energy () for each system, but also due to the additional dependencies of the functions  and ***Q*** on **θ**. For the parameter estimation, we avoid this added complexity by fixing the molecule to the crystalline conformation determined in the DFT-D reference data (**θ**^*ref*^), and only minimize the *U*_*inter*_ component over the remaining rigid-body geometric variables (**Ω**). In effect, we assume that the DFT-D crystalline conformation and its corresponding Δ*U*_*intra*_ would be accurately predicted by our HAIEFF model. This is not unreasonable given that in a flexible minimization, these intramolecular terms would be determined by similar DFT calculations (albeit with different levels of theory and basis sets). Moreover, given that the DFT-D reference energies can be quite easily decomposed into *U*_*inter*_ and Δ*U*_*intra*_, this approach is prudent. The values of the geometric variables at the optimal crystal geometry are denoted  and are determined as the arguments of a local minimization (arg min) of the crystal structure at fixed conformation as shown in Problem (LM).
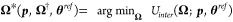
LMwhere the starting values of the geometric variables are denoted . A suitable choice for the starting point is important as the lattice energy function is highly nonconvex and the solution of the optimization () depends strongly on the starting point used (). For the optimization of crystal structures in our parameter estimation, it is usually sufficient to start the optimization at the DFT-D determined reference geometry, denoted  (i.e., ). An important characteristic of Problem (LM) is that the optimal geometric variables depend implicitly on the parameter values used in the model, as well as the initial starting point. The optimal intermolecular energy (), which is required within the parameter estimation, is calculated directly at the optimal geometry identified in Problem (LM):
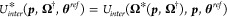
6

Additional dependencies of the optimal geometric variables and energies are not listed explicitly for clarity, but they include the choice of electrostatic model (point-charges or multipoles) as well as the optimization algorithm and algorithmic parameters used in the local minimization.

### 2.2 Parameter Estimation Methodology

For the lattice energy model given in Section 2.1 to be effective, the minimization of the lattice energy function should reliably reproduce the geometry of experimental crystal structures, as well as their overall binding energy. The quality of the model predictions are fundamentally dependent on the quality of the physics-based and empirical parameters supplied to the model. To assess the quality of a prediction, a merit function is required. The form of the merit function is derived based on the work of Gatsiou^38^ and Gatsiou et al.^34^ and is formulated in terms of geometry and energy deviations (residuals) for a set of optimized crystal structures compared to a set of reference structure energies and geometries.

To define the geometry contribution of the merit function, a set of geometry residuals, ***G***_*s*_, for structure *s*, are determined from deviations in unit cell parameters and fractional atomic coordinates in the asymmetric unit cell. The residuals relating to unit cell geometry (denoted by the vector ***X***_*s*_) include lattice variables (cell lengths and angles) which are independent and are unconstrained as a result of space group symmetry. The lattice variables are a subset of the geometric variables (i.e., ). The number of such unit cell parameters is denoted  and is dependent on the lattice type (e.g., triclinic, monoclinic, etc.) of the crystal. These residuals are measured as *relative* deviations from the reference structure and normalized by the square root of the number of residuals of this type,
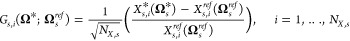
7

For deviations in the fractional coordinates of molecules in the asymmetric unit cell, the geometry and energy of the crystal structure can be invariant to certain translations of the entire asymmetric unit cell in one or more fractional coordinate directions, subject to space group symmetry. To ensure that these degenerate geometric representations are evaluated identically, fractional coordinates are measured *relative* to a reference atom in the asymmetric unit cell. This is arbitrarily taken as the first atom listed in the asymmetric unit cell.  denotes the *total* number of atoms in the asymmetric unit cell (including atoms from other molecules if ). The vector of all fractional coordinates in the asymmetric unit cell, , contains  elements for the fractional coordinates of each atom in 3 dimensions, , , and . These are used to calculate the vector ***Y***_*s*_ of  elements containing relative fractional atomic coordinates:
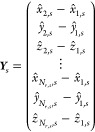
8In crystals where , the deviations in atomic positions for all asymmetric molecules are taken with respect to the first atom in the asymmetric unit cell. Geometry residuals with respect to the relative fractional coordinates are taken as unscaled deviations in the elements of ***Y***_*s*_ from the reference structure geometry, normalized by the square root of the number of residuals of this type (),
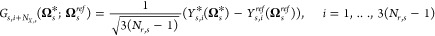
9and thus the geometry residual vector for structure *s*, ***G***_*s*_, contains  components.

One energy residual is included per structure and it is formulated as a *relative* deviation from the reference intermolecular energy of that structure ().
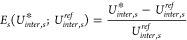
10

The merit function of structure *s* is denoted by *MF*_*s*_ and is calculated as a weighted sum-of-squares of the geometric and energetic residuals,
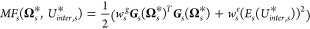
11where  and  denote weighting factors for structure *s* that are used to tune the trade-off between geometric and energetic contributions in the merit function. Some arguments have been omitted for clarity. While “1/2” prefactor will not affect the results of minimizing *MF*_*s*_, its inclusion simplifies the subsequent analytical gradient expressions and also provides a parallel to the conventional form of a maximum likelihood estimation.

Summing *MF*_*s*_ over all structures, , where *NS* is the number of structures in the parameter training set, forms the Total Merit Function (*TMF*). For a subset of the empirical parameters to be optimized , optimal parameter estimates, , are obtained by solving Problem (PE).
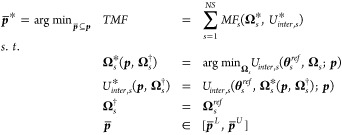
PEwhere  and  denote the vectors of lower and upper bounds, respectively, for the optimized parameters. For our purposes,  are simply constrained to be non-negative (, ).

#### 2.2.1 Derivatives with Respect to Parameter Values

Nesting of the lattice minimization within the larger parameter estimation framework shown in Problem (PE) results in a bilevel programming problem: an inner level involving the rigid lattice energy minimization of each structure with respect to  and an outer level involving the *TMF* minimization with respect to . This bilevel structure presents a significant challenge to solving the parameter estimation problem for a number of reasons. One of the most challenging aspects of this optimization problem is the calculation of derivatives of each merit function with respect to the parameter values. Gradients are typically obtained numerically using finite differences, such that the lattice energy minimization problems must be resolved for each finite difference step.^7,33,34^ For instance, using second-order centered finite differences, gradient calculations would require  lattice minimizations per set of gradient calculations, where *NP* is the number of parameters being optimized. This drastically increases the computational cost of the entire approach as *NP* and/or *NS* are increased. Finite differences are also subject to much more numerical noise than analytical calculations. This can lead to inaccuracies in the calculation of gradients and premature convergence or failure of typical optimization algorithms.

Instead, we seek to derive analytical relations for the gradients of the *TMF*. Differentiation of *MF*_*s*_ as defined in eq 11 with respect to an estimated parameter  results in the following expression:
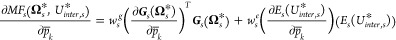
12where .  and  are algebraic functions of  and , respectively, but  and  are obtained from the solution of a local minimization and do not have a closed form expression. Nevertheless, analytical expressions for the derivatives of these variables with respect to  can be obtained by manipulation of the optimality conditions of the lattice minimization defined in Problem (LM). The full details the derivations can be found elsewhere.^11^ An outline of the derivations along with the final equations are supplied here.

To obtain derivatives of the optimal intermolecular energy of structure *s* with respect to , we differentiate eq 6 with respect to  taking note of the multiple dependencies of the optimal intermolecular energy function on . Through the stationarity conditions imposed by the lattice energy minimization, it can be shown that the derivatives may be expressed as in eq 13.
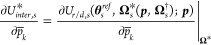
13This is a remarkably simple expression which relates the change in optimal intermolecular energy to the derivative of the repulsive/dispersive energy contribution evaluated at the optimal crystal geometry. This is despite the additional indirect dependence of  on the parameter values through . The required derivatives of the energy residual,  in eq 12, can be shown to be directly proportional to this derivative.

Acquiring the derivatives of the geometry residuals is more involved, but ultimately reduces to determining the first derivatives of the optimal geometric variables, , with respect to parameters  (). To determine this, we differentiate the optimality conditions imposed by Problem (LM) with respect to the variables in . After simplification, this results in eq 14.
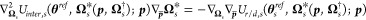
14 is the second-derivative Hessian matrix of the intermolecular energy function with respect to the optimization variables , while  is a vector of second-order cross derivatives of the repulsive/dispersive energy component with respect to optimization variables  and . Once the lattice energy minimization is complete, both of these matrices can be evaluated. The former is independent of the parameter derivatives and thus can be reused for all parameters in the set of variables . Evaluation of the latter utilizes the first-derivatives and second-order cross derivatives with respect to the parameter values for each parameter variable. Crucially, analytical expressions for all of these derivative terms have been explicitly derived in ref (11). As such, these quantities are likely to be far cheaper to evaluate than using finite difference calculations around a lattice minimization, and also benefit from less numerical noise than finite differences. Moreover, no additional lattice minimizations need to be carried out since all of the required quantities are defined at the optimal structure geometry obtained for the evaluation of the merit function. With the values of the matrices determined,  can be obtained by solving the set of linear equations formed by eq 14.

The effects of combining rules on the parameters , , and  can also be enforced through the derivatives of the merit function by applying the chain rule of differentiation. For instance, if the parameter  depends on the parameter  through a combining rule relationship (i.e., ), then the derivatives of  can be modified to include this additional dependence through eq 15.
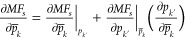
15where differentiating the combining rule relationships specified in eq 5 leads to the following equations:
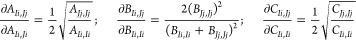
16

Overall, the use of analytical relations for evaluating the derivatives of the merit function should significantly reduce the computational cost of optimizing the total merit function, while also improving the precision with which parameter estimates can be obtained. The main barrier against implementation of this approach is that many quantities necessary for the calculation, such as the Hessian matrix or the derivatives of intermolecular energy with respect to the parameter values in eq 14, cannot be easily obtained or implemented in any existing software for lattice minimizations. Even when such quantities could be feasibly obtained, the quantities may only be calculated approximately using finite differences. To reap the benefits that these calculations may bring necessitates the development of new tools for performing intermolecular energy calculations and lattice minimizations.

## 3 Algorithms and Implementation

### 3.1 Crystal Structure Optimizer—Rigid Molecules

To implement the parameter estimation methodology outlined in Section 2.2 an appropriate tool for calculating intermolecular energy is required. While several programs are available for the calculation of the intermolecular energy and its derivatives, no such programs support the calculation of all the additional quantities needed by our methodology. In aid of this, the code Crystal Structure Optimizer—Rigid Molecules (CSO-RM) has been developed for the calculation of the intermolecular energy and its derivatives, and for the minimization of the lattice energy.^11^

CSO-RM uses a HAIEFF model to calculate intermolecular interactions. The form of the intermolecular model is the same as that given in Section 2.1, comprising electrostatic and repulsion/dispersion contributions. The repulsion/dispersion calculation is implemented as specified in Section 2.1, although additional pairwise potential functions are also supported. The electrostatic contribution is calculated as the sum of interacting atom-centered multipole expansions. All possible multipole interactions up to a combined rank of  are implemented in the code, where *l*_1_ and *l*_2_ are ranks of interacting multipoles on each atom site.^57^ The specific case of a point-charge based electrostatic model is obtained by limiting the calculation to only interactions where . To ensure that the summation of multipole interactions is calculated accurately, a generalized Ewald summation is employed based on the work of Nijboer and De Wette^58^ and Vasileiadis^59^ with additional modifications to improve the speed of the calculation. Unlike programs such as DMACRYS,^60^ which employs Ewald summations for low-order multipole interactions and direct space summations for higher-order interactions, CSO-RM uses Ewald summations for multipole interactions of arbitrary rank. This ensures that high accuracy can be achieved for both conditionally convergent and slowly converging summations at minimal computational expense. Using this model, the intermolecular energy can be calculated along with the following analytical derivative quantities: first and second derivatives with respect to the rigid-body variables (**Ω**); first derivatives with respect to the parameters of the repulsion/dispersion contributions (***p***); and cross-second derivatives with respect to the rigid-body variables and the parameters of the repulsion/dispersion contributions (**Ω** and ***p***).

An optimization algorithm for performing lattice energy minimizations is also implemented within the CSO-RM software library. The algorithm follows a two-stage optimization procedure: 1) Starting from the initial vector , the intermolecular energy is locally optimized using the quasi-Newton optimization algorithm implemented in the NAG E04UFF algorithm,^61^ and 2) following initial optimization of the crystal structure in step 1, the estimate of  is refined using a modified-Newton optimization algorithm based on the analytical second derivatives of intermolecular energy. We find that this second stage is important when extremely high precision in the estimates of  is required. This is the case when iterations are taken around the lattice minimization, as performed in our parameter estimation methodology. Space group symmetry constraints are employed during the optimization, and molecules in the asymmetric unit cell are assumed to be rigid.

CSO-RM is implemented in Fortran90 as a library of transferable software components. This allows programs to readily interface with CSO-RM for applications in parameter estimation and beyond, and to avoid loss of numerical precision during calculations. This is essential if high precision is required. For more details on the algorithm, implementation, and testing of CSO-RM, see the work of Bowskill.^11^

### 3.2 CrystalEstimator

To solve Problem (PE), we have developed the CrystalEstimator program which leverages gradient-based optimization algorithms to solve the outer problem. CrystalEstimator interfaces with CSO-RM to carry out the inner minimizations. Multistart capabilities are integrated into CrystalEstimator through a quasi-random initialization sequence^62^ in order to increase the probability of finding the global optimal solution of this highly nonconvex problem. However, there are a several problem-specific complications that require more-bespoke solutions.

- **Discontinuities in the objective function**

A complication of the parameter estimation is that the bilevel structure of Problem (PE) introduces discontinuities into the total merit function.^38^ One possible explanation for this is the fact that the crystal intermolecular energy surface is innately nonconvex and can contain many minima with distinct crystal energy/geometry. As the outer optimization alters the parameter values, the inner optimizations may converge to different minima as the contours of the energy surface (and the minima present on the surface) change. If the optimized crystal energy and/or geometry change dramatically because of this, it will manifest as discontinuities in the *TMF* solution surface.

- **Lattice energy minimization failures**

During the course of the optimization algorithm it may not be possible to solve one or more of the lattice energy minimizations specified in Problem (LM). The lattice energy minimization may converge to a saddle point or may fail to converge tightly to a locally minimum point. This commonly happens when the parameter estimates are poor and the resulting pairwise Buckingham potential displays nonphysical behavior at short intermolecular contact distances.

The algorithm and strategies to address these challenges are described in the remainder of this section.

#### 3.2.1 CrystalEstimator Algorithm Overview

**Figure 1:**
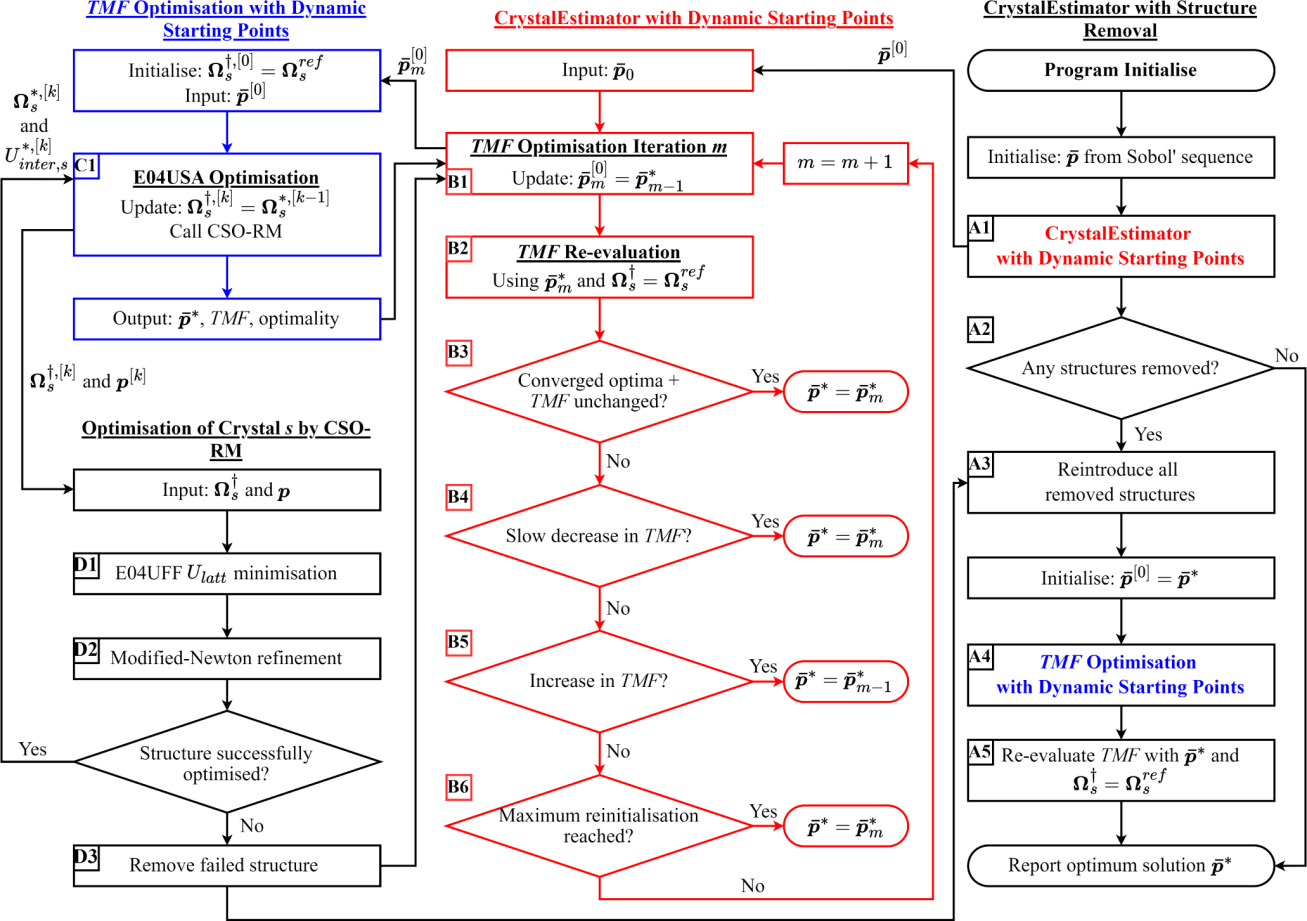
Schematic of the CrystalEstimator algorithm with dynamic starting points, algorithm reinitialization, and temporary structure removal. The symbols associated with arrows show the transfer of information between stages. Key stages are enumerated and detailed within the main text.

The CrystalEstimator Algorithm is illustrated in Figure 1. In its most basic form, CrystalEstimator will minimize the *TMF* from a starting vector of parameter estimates, , using the E04USA quasi-Newton optimization algorithm as implemented in the NAG library.^61^ At each major iteration of the *TMF* optimization (*k*), any required function and/or gradient evaluations of the *TMF* are determined with corresponding calls to CSO-RM (D1 and D2 in Figure 1).  is altered based on the gradient information or the value of the objective function and the process is repeated until optimality is reached, as assessed by the E04USA subroutine. On top of this basic implementation, several key adaptations have been added to manage the aforementioned challenges.

First, to suppress discontinuities in the objective function we introduce the concept of dynamic starting points to the *TMF* optimization. The reference structure geometries are used as the starting points for the lattice energy minimizations in only the first *TMF* evaluation by E04USA (). As the sequence of iterates proceeds, these starting points are dynamically updated to the optimal geometry obtained in the preceding *TMF* evaluation such that in iteration *k*,  (C1 in Figure 1). For small steps in the parameter values, this approach significantly reduces the possibility of discontinuities manifesting in the *TMF* as the same minimum is far more likely to be identified in successive *TMF* evaluations. An exception to this arises in the case where two minima coalesce on the intermolecular energy hypersurface. When this occurs, the stationary point corresponding to the shallower minimum (higher *U*_*inter*_ value) transitions through a saddle point for some intermediate parameter values, and eventually the two wells combine to produce a single well. This is unfortunately an unavoidable consequence of the formulation of Problem (PE).

A consequence of using dynamic starting points is what we define as starting point “drift”. As the starting points for the lattice energy minimizations are updated at each *TMF* evaluation, the starting point may drift over time and eventually may no longer reside in the immediate proximity of the reference structure geometry. To ensure that the final *TMF* value being evaluated is congruent with Problem (PE), it is important that following termination of E04USA, the *TMF* is re-evaluated using the original reference structure geometries as starting points (B2 in Figure 1). That is, if E04USA terminates after *k* major iterations, a *TMF* evaluation is conducted starting from the reference geometries. If the NAG algorithm converges to a local minimum and the *TMF* is unchanged upon its re-evaluation, it can be inferred that any effects of starting point drift are minor and an optimum to Problem (PE) has been found. CrystalEstimator will accept this as the final set of parameter estimates (B3 in Figure 1).

In large optimization problems, it is possible for either the E04USA algorithm to be unsuccessful in converging to a local minimum and/or the *TMF* changes significantly upon re-evaluation. In this case, another iteration of *TMF* optimization is undertaken, where in the *m*^*th*^ iteration, the vector of parameter values is initialized at the set of parameter estimates obtained in the  optimization () (B1 in Figure 1). However, prior to this, three additional termination criteria are evaluated (B4 to B6 in Figure 1). First, if the re-evaluated *TMF* value in the *m*^*th*^ and  optimization attempt decreases, but by an insufficient value, CrystalEstimator terminates and accepts the parameter estimates determined in the *m*^*th*^ iteration. It is likely that the solution surface is relatively “flat” near the optimum parameter values hence any additional attempts to reoptimize the *TMF* will yield little improvement. Second, if the re-evaluated *TMF* value increases in the *m*^*th*^ iteration compared to the  attempt, CrystalEstimator terminates and accepts the parameter estimates determined in the  iteration. This again indicates that the parameter estimates are close to the true optimum and we sensibly choose the best available solution. Although these criteria do not resolve the failure of E04USA to converge to an optimum, it must be stressed that the objective of (PE) is to minimize the *TMF* when . With dynamic starting points, E04USA is not directly solving Problem (PE), but rather driving the parameters in the required direction. From this perspective, the convergence of the re-evaluated *TMF* value is more crucial. The final convergence criteria which CrystalEstimator considers is whether the user-specified reinitialisation limit has been reached. If none of these secondary termination criteria are met, the *TMF* is optimized again. Because the first two of these criteria (B4 and B5 in Figure 1) require comparison to the *TMF* in a preceding iteration, they are naturally skipped in the *m* = 0 iteration where only the third criteria is assessed. In this manner, *TMF* optimizations are reattempted until one of the four convergence criteria are satisfied. In each iteration, the dynamic starting points are reinitialised such that the first *TMF* evaluation by E04USA uses .

As described earlier, while progressing toward the optimum parameter estimates, intermediate parameter values may prove ill-suited for the lattice energy minimization of some structures in the training set. This results in failed lattice energy minimization and no proper way to evaluate the residuals associated with those structures. The algorithm could continue despite this with the introduction of a penalty to the objective function, but, through testing, we find this tends to lead to unsatisfactory progress in the optimization. Our strategy for handling lattice minimization failures is therefore to remove the problematic structure(s) from the training set and reinitialize the parameter estimation algorithm (D3 in Figure 1). When a failure is encountered, the *TMF* optimization is aborted and the failed structure is temporarily removed from the training set. The algorithm is then reinitialized with the reduced training set and the optimization is restarted from the parameter estimates at the last iteration. This allows the optimization to “escape” from regions of the parameter space where the lattice minimization cannot be reliably solved for certain structures. On the basis of the remaining structures, CrystalEstimator will proceed until one of the previously described termination criteria (B3 to B6) are met. Following this, any removed structures are reintroduced into the training set and a final *TMF* optimization and *TMF* re-evaluation are conducted (A3 to A5 in Figure 1). This optimal result is reported without any possibility of further iterations of *TMF* optimization. It should be noted, that the dynamic starting point and structure removal strategies are fully modular, and in principle one could choose to enable any combination of these strategies with the basic CrystalEstimator algorithm.

#### 3.2.2 MPI Parallelization

Due to the complexities of lattice sum calculations, the most costly aspects of the algorithm are the lattice minimizations of structures in the training set, which are needed to obtain  and , and the calculation of the derivatives of the residuals. Because the lattice minimization and residual calculations for each structure are independent from one another, this enables their parallelization. Parallelization is implemented in CrystalEstimator using Message Passing Interface (MPI), where  computing cores are distributed into  workers and 1 bookkeeper. The CrystalEstimator code can also support serial operation (i.e., ) where all calculations are performed on a single processor with no bookkeeper.

In parallel operation, the workers initially load all training set data into memory and then remain idle until communicated to by the bookkeeper with instructions. Each worker is then assigned a single structure by the bookkeeper for processing. The parameter values at the current iteration, a structure, as well as the starting point and initialization data for the lattice minimization are passed to the worker. The worker performs a lattice minimization and, if requested by the bookkeeper, may also calculate the gradients of the residuals for the specified structure. The residuals and the residual gradients are then returned to the bookkeeper with the optimal structure geometry and other meta-data.

The bookkeeper initializes and executes the E04USA algorithm and at each iteration distributes the structure calculations among the workers one at a time along with the parameter values at the current iteration and all additional information required for the optimization. Once all work has been distributed, the bookkeeper waits for communication from the workers. After contact from a worker, the bookkeeper receives the values of the calculated residuals and various other meta-data, and assigns the worker a new structure for processing. This is continued until the training set has been exhausted and the residuals have been calculated for all of the structures. After the first iteration of the algorithm, computational cost estimates are made for each structure calculation based on the CPU times obtained in the previous iteration. The structures are then supplied to the workers in order of decreasing estimated computational cost. This ensures that the most costly minimizations and gradient calculations are initiated first to limit overall idle time for the workers. Once the optimization has terminated, the bookkeeper informs all workers to also terminate and all processes can exit the program.

#### 3.2.3 Global Optimisation

Upon successful completion, the optimization procedure described so far results in the identification of only a local minimum. The total merit function is nonlinear and may exhibit multiple minima, or could be subject to discontinuities at or around the optimal solution of Problem (PE). To thoroughly sample the search space of possible parameter values, multiple instances of the parameter estimation algorithm are run from different parameter starting vectors, . Each starting vector is generated from Sobol’ sequences^62,63^ within the specified bounds on the parameter values, with the Sobol’ sequences ensuring that the search space is covered as efficiently as possible for any number of sampling points. Due to the deterministic nature of Sobol’ sequences, *post hoc* addition of starting points can be done without risk of deteriorating the effectiveness of the sampling.

Each of the Sobol’ point optimizations can be performed completely independently and run simultaneously across high performance computing clusters. At termination, the optimal value of the total merit function and the associated parameters are reported as the solution obtained from each Sobol’ seed used to generate . Post-analysis of the results is carried out by comparison of the optimal total merit function values and solutions are clustered into distinct minima. The parameter values which produce the lowest total merit function can then be selected as the optimal parameter set.

## 4 Reference Datasets and Test Cases

### 4.1 Generation of Reference Datasets

When synthetic data are used to fit potentials, it is desirable to generate that data using models of maximum accuracy which remain affordable for a given availability of computational resources. We choose to employ solid-state DFT-D calculations for the generation of reference data for a few reasons. For this application, DFT-D offers a competitive balance between accuracy and cost, relative to other high accuracy *ab initio* methods. As alluded to before, most modern CSP workflows utilize DFT-D as a final refinement tool, making it a rational choice of “ground truth”. Finally, calculations using DFT-D can be performed quite routinely using modern software packages. To ensure that the datasets exhibit broad chemical diversity, we generate them in two steps. First, a primary dataset is constructed by searching the CSD for experimental structures using relatively strict search criteria. These include restrictions on molecular size, molecular rigidity, and the completeness of the reported geometric data. A secondary dataset of molecular crystals is subsequently curated in order to supplement data for atomic environments which are otherwise absent or scarce in the primary dataset (e.g., molecules simultaneously containing Cl and S).

#### 4.1.1 Solid-State DFT-D Computations

All solid-state DFT-D calculations are performed using the Vienna *ab initio* software package (VASPv5.4.4).^42^ The TPSS functional and D3 dispersion correction are selected due to their good performance in multiple benchmark studies alongside PAW pseudo-potentials. A large energy cutoff of 1000 eV is used with a tight Γ-centered *k*-point mesh of at most  Å^–1^ reciprocal-distance between each *k*-point. The crystal structures are relaxed allowing variation of the unit cell lengths and angles, and atom positions until all forces are less than 0.01 eV Å^–1^. After the initial convergence of each DFT-D optimization, calculations are restarted, sometimes multiple times, to mitigate the effects of Pulay stresses until the optimal geometry and energy converge. To obtain the reference energy values, it is necessary to also evaluate the monomer energies for all molecules in the asymmetric unit cell. From the relaxed crystal structure, each distinct molecule, *m*, in the asymmetric unit cell is placed in a fixed cubic unit cell with a minimum intermolecule contact distance of at least 16 Å. It is assumed that, at this separation, intermolecular interactions are negligible. A single-point calculation is conducted to obtain the monomer energy at the crystalline conformation () while an optimization is conducted to obtain the corresponding energy of the monomer *in vacuo* (). The desired energy values for a crystal structure can then be obtained as,
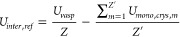
17
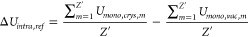
18
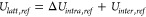
19where *U*_*vasp*_ is the optimal crystal energy reported by VASP, while , , and  are the intermolecular, intramolecular and lattice energy of the crystal derived from the VASP calculations. *Z* and *Z*^′^ correspond to the number of molecules in the unit cell and asymmetric unit cell, respectively. From the output of VASP, the symmetry relations for each of the optimized structures are recalculated using PLATON.^64^ In some cases, the symmetry of the crystal cannot be reduced to that of the experimental structure, in which case the system is treated as .

#### 4.1.2 Generation of Primary Datasets

As a first pass for data generation, candidate structures are obtained from two sources. First, structures are taken from the parameter fitting work of Gatsiou et al.,^34^ with the omission of structures containing linear molecules including acetylene (ACETYL05), cyanogen (CYNGEN), and dicyanoacetylene (DCYANM), as their effective treatment would introduce additional complexities in the structure optimization code. The primary reference dataset is then supplemented with a large number of additional structures taken from the CSD to extend the size of the existing dataset and develop new datasets for the fitting of additional atom–atom interactions. To fit many of the interactions commonly present in organic compounds, we construct reference datasets with molecules containing carbon, hydrogen, nitrogen, oxygen, fluorine, chlorine, and sulfur. Only structures with no known disorder and with all atom positions recorded in the crystallographic file are added to the reference datasets. No constraints are enforced as to the experimental methods used, the discrepancy factors in structure resolution, or the temperature at which the crystal structures were resolved. The use of DFT-D calculations to optimize the geometry of these structure should eliminate any small discrepancies arising from measurement errors or temperature effects. In general, crystals comprising few small rigid molecules in the unit cell are preferentially selected to reduce the cost of the DFT-D calculations. This may lead to some biases in the reference datasets toward conjugated or aromatic systems which occur more commonly. Systems with several polymorphic structures matching the above criteria are also preferentially selected to allow the assessment of the model predictions of relative energy differences between polymorphs. Only structures for which the final DFT-D optimized geometry produce a good match with the experimental geometry are included in the reference set. This excludes the structure of formaldehyde (KEMZIL), among others, due to large deviations between the DFT-D optimized and experimental structure.

A summary of the primary reference datasets is given in Table 1. In total, the datasets contain 445 structures which can be broadly split into 313 non-hydrogen bonding (RS1–RS23) and 132 hydrogen bonding structures (RS24–RS34, indicated by the presence of either O–H or N–H bonds). Structures are selected to contain diverse chemical environments with close contacts present for each atom–atom interaction with the exception of sulfur−chlorine interactions which are absent from the reference datasets.

**Table 1:** List of Primary Reference Datasets Derived from DFT-D Computations

Reference Set	Typical compound classes	Atom Types Present	Number of structures
RS1	Aromatic/Aliphatic hydrocarbons	C, H	18
RS2	(3^◦^) Amines/Imines/Nitriles/Azo-groups	C, H, N	30
RS3	Ethers/Aldehydes/Ketones/Esters	C, H, O	30
RS4	Chlorinated hydrocarbons	C, H, Cl	25
RS5	Fluorinated hydrocarbons	C, H, F	10
RS6	Thioethers/Disulfides/Thiocarbonyls	C, H, S	16
RS7	RS2/RS3/Nitro-groups	C, H, O, N	18
RS8	RS2/RS4	C, H, Cl, N	17
RS9	RS3/RS4	C, H, Cl, O	13
RS10	RS2/RS5	C, H, F, N	12
RS11	RS3/RS5	C, H, F, O	9
RS12	RS4/RS5	C, H, Cl, F	6
RS13	RS2/RS6	C, H, S, N	16
RS14	RS3/RS6/Sulfoxides/Sulphones	C, H, S, O	16
RS15	RS5/RS6	C, H, S, F	5
RS16	RS4/RS7	C, H, Cl, N, O	10
RS17	RS5/RS7	C, H, F, N, O	13
RS18	RS2/RS4/RS5	C, H, Cl, F, N	7
RS19	RS3/RS4/RS5	C, H, Cl, F, O	6
RS20	RS2/RS14	C, H, S, N, O	14
RS21	RS2/RS5/RS6	C, H, S, N, F	8
RS22	RS5/RS14	C, H, S, O, F	9
RS23	RS2/RS3/RS4/RS5	C, H, Cl, F, N, O	5
RS24	RS2/(1^◦^/2^◦^) Amines	C, H, N, N–H	18
RS25	RS3/Alcohols/Carboxylic Acids	C, H, O, O–H	15
RS26	RS7/RS24/RS25/Amides/Oximes	C, H, N, O, N–H, O–H	21
RS27	RS3/RS4/RS24	C, H, N, O, N–H, Cl	15
RS28	RS2/RS4/RS25	C, H, N, O, O–H, Cl	11
RS29	RS4/RS26	C, H, N, O, N–H, O–H, Cl	2
RS30	RS3/RS5/RS24	C, H, N, O, N–H, F	15
RS31	RS2/RS5/RS25	C, H, N, O, O–H, F	13
RS32	RS14/RS24	C, H, N, N–H, S	9
RS33	RS14/RS25	C, H, O, O–H, S	8
RS34	RS14/RS26	C, H, N, O, N–H, O–H, S	5
Total			445

Reference sets RS1 to RS6 are the most fundamental reference datasets consisting generally of carbon, hydrogen and one other element (except for RS1 which includes only aliphatic and aromatic hydrocarbons). RS2 introduces a number of common nitrogen environments such as tertiary (3°) amines, imines, nitriles, and azo-groups. It is worth pointing out that the definitions used here for many of the functionals groups, such as amines and imines, are determined based on the atoms they are bonded to and irrespective of whether the atom appears in an aromatic ring. This broadly encompasses other common functional groups within these definitions, including many different classes of heteroaromatic systems which may be considered distinctly different by an organic chemist. Common oxygen environments such as ethers, aldehydes, ketones, and esters are considered in RS3. RS4 and RS5 extend into chlorinated and fluorinated hydrocarbon systems, respectively, where the halogen atom is substituted onto either an aromatic or methyl carbon atom. Finally, RS6 incorporates various sulfur environments, including thioethers, disulfides, and thiocarbonyls. The remaining reference sets in the non-hydrogen bonding reference datasets (RS7–RS23) include combinations of the functional groups found in RS2-RS6. Some additional functional groups are also introduced such as nitro-groups which first appear in RS7, as well as sulfoxides and sulfones which first appear in RS14.

For the hydrogen bonding reference datasets, RS24 introduces organic compounds containing N–H bonds. These mainly include primary/secondary (1°/2°) amines. RS25 introduces organic compounds with O–H bonds, including alcohols and carboxylic acids. The remaining reference datasets once again use various combinations of the functional groups from the non-hydrogen bonding and hydrogen bonding sets to fit cross-interactions. Additional functional groups such as amides or oximes can also be found in the later reference datasets (RS26–RS34).

#### 4.1.3 Generation of Atom Environment Datasets

While the primary datasets attempt to capture a wide variety of atomic environments by including a mix of “typical compound classes”, this is mostly based on subjective judgment, and no quantitative measure of “diversity” was actively considered during its construction. As such, following the initial pass of data generation, a more detailed analysis of the atomic environments is conducted. Rather than characterizing the atomic makeup of the crystal structures only by element, each element is further distinguished by more descriptors, including the atom’s coordination number, valency, and immediate bonded neighbors. Because these descriptors are conveniently described by SMARTS notation (Table 2), this facilitates use of the CCDC’s Python API^65^ for analysis of the existing datasets and during the subsequent candidate structure search.

**Table 2:** Description of the Atom Types Used for Analysis of Diversity in the Reference Dataset^a^

Atom	SMARTS	Description	Chemical Environment
C_2,*,*_	[#6X2]	Doubly coordinated C	sp carbons
C_3,*,*_	[#6X3]	Triply coordinated C	sp_2_ carbons
C_4,*,*_	[#6X4]	Quadruply coordinated C	sp_3_ carbons
O_1,*,*_	[#8X1]	Singly coordinated O	Carbonyls/Esters/Carboxylic acids
O_2,*,*_	[#8X2]	Doubly coordinated O	Hydroxyls/Ethers,/Esters/Carboxylic acids/Oxazoles
N_1,3,*_	[#7X1v3]	Singly coordinated N, valency 3	Nitriles
N_2,3,*_	[#7X2v3]	Doubly coordinated N, valency 3	Aromatic nitrogens
N_3,3,*_	[#7X3v3]	Triply coordinated N, valency 3	Amines/Amides
N_3,5,*_	[#7X3v5]	Triply coordinated N, valency 5	Nitros
F_*,*,*_	[#9X1]	Singly coordinated F	Regular fluorines
Cl_*,*,*_	[#17X1]	Singly coordinated Cl	Regular chlorines
S_*,*,O_	[#16]=[#8]	S double-bonded to O	Sulfoxides/Sulfones/Sulfonamides
S_1,*,*_	[#16X1]	Singly coordinated S	Thiocarbonyls/Thioamides
S_2,*,*_	[#16X2]	Doubly coordinated S	Thiols/Sulfides/Disulfides/Thioesters/Thioazoles
H_*,*,N_	[H][#7]	H bonded to N	Amines/Amides
H_*,*,O_	[H][#8]	H bonded to O	Hydroxyls
H_*,*,S_	[H][#16]	H bonded to S	Thiols
H_*,*,C_	[H][!#7;!#8;!#16]	H not bonded to N, O, or S	All other Hs must be carbon-bonded

a Each atom label begins with its element followed by three subscripts corresponding to the coordination number, valency, and the bonded-neighbor element respectively. “*” represent wildcards and indicate that a feature is disregarded in the atom environment definition. Using the CCDC’s Python API, presence of these atomic environments in a molecule can be queried using the corresponding SMARTS string.

Because the force-fields we aim to parametrize involve pairwise atomic interactions, we consider the number of structures that contain each pair of atoms. This generates a matrix that quantifies the representation of pairwise interactions in our datasets and allows us to focus our data generation efforts toward structures containing atom types or atom pairs that are sparse. The criteria applied during the construction of the primary reference sets are also enforced during this secondary search of the CSD, using the relevant property metrics accessible through the Python API. The size of each candidate molecule is constrained based on the total number of atoms in a structure, while flexibility is limited by the total number of sp_3_ carbons present in a structure. Structures with  and  are preferentially searched for, and disordered structures are rejected. Any structure that is already in the primary datasets is rejected to avoid multiple instances of the same crystal structure, although polymorphic forms are accepted. If the desired number of candidates cannot be identified for a given atom type or pair, some of these criteria are relaxed slightly to expand the search space. As before, no constraints are placed on the method used to resolve the crystal geometry. Unlike the primary search, we do not limit ourselves to structures that have *all* atom positions resolved. Instead, structures are considered as long as they have all *heavy*-atom positions identified. Any missing hydrogens can be placed in standard positions using Mercury’s in-built molecule editor. Some structures that require this treatment include 2-mercapto-pyridine-4-carboxylic acid (MPYDCY) and cis-2-butene-episulfone (BUTSUL10). This approach is deemed acceptable since hydrogen positions determined by experimental methods are generally considered to be not very accurate. Moreover, when the DFT-D optimized geometries are compared to their experimental counterparts, the CCDC’s COMPACK tool,^66^ by default, ignores hydrogen positions. The list of candidates generated through the Python API undergoes a final round of manual screening in order to filter out highly irregular structures or structures that are likely to exhibit extreme flexibility. Despite the limits imposed on the number of sp_3_ carbons, excessive flexibility can still occur if all the permitted sp_3_ carbons are directly bonded to each other in a linear fashion. The finalized list of structures then undergoes the same treatment as the primary reference datasets.

The atom environment datasets that are generated in this way are summarized in Table 3. The first few atom environment groups (AE1-AE6) are defined quite loosely, in many cases encompassing all definitions for a given element. This “shotgun” approach is initially attempted in order to more quickly fill large voids in the atomic environment matrix. Thereafter, we narrow our search to more specific atom pairs. Intuitively, the atomic environments most sparsely populated in the primary datasets are those involving sulfurs and the halogens since these elements are less common in the CSD. Additionally, since the primary datasets have a greater proportion of structures that are non-hydrogen bonding, the atom environment datasets place more focus on hydrogen bonding structures containing hydrogens bonded to either oxygen or nitrogen. Both of these trends are self-evident from the selection of the target environments used in AE7-AE18. Crucially, the sulfur−chlorine interaction which was completely omitted in the primary datasets has been introduced here. Finally, readers might perceive an “asymmetry” in the search criteria applied for AE15 and AE16. The lack of corresponding “S_1,*,*_” searches is not an oversight. Searches were attempted for “S_1,*,*_ + F_*,*,*_”, “S_1,*,*_ + Cl_*,*,*_”, and “S_1,*,*_ + S_*,*,O_”, but these searches did not yield a sufficient number of viable candidates and thus the search candidates are not included.

**Table 3:** List of Atom Environment Reference Datasets Obtained from DFT-D Computations

Reference Set	Target Atomic Environments	Number of Structures
AE1	Any carbon but preferentially C_2,*,*_	14
AE2	S_*,*,O_/S_1,*,*_/Thiols	21
AE3	S_*,*,O_	12
AE4	Any nitrogen	17
AE5	Any oxygen	10
AE6	Nitro groups (N_3,5,*_ + O_1,*,*_)	8
AE7	S_1,*,*_ + H_*,*,N_	12
AE8	S_1,*,*_ + H_*,*,O_	10
AE9	S_2,*,*_ + H_*,*,N_	13
AE10	S_2,*,*_ + H_*,*,O_	16
AE11	S_*,*,O_ + Any nitrogen + Preferentially with H_*,*,N_	23
AE12	S_*,*,O_ + Any oxygen + Preferentially with H_*,*,O_	10
AE13	S_*,*,O_ + Any halogen	25
AE14	H_*,*,O_ + Any nitrogen	61
AE15	S_2,*,*_ + Cl_*,*,*_	14
AE16	S_2,*,*_ + S_*,*,O_	12
AE17	Cl_*,*,*_ + H_*,*,O_ or H_*,*,N_	15
AE18	F_*,*,*_ + H_*,*,O_ or H_*,*,N_	17
Total		310

A compiled list of all structures included in the reference datasets (primary and atom environment) can be found as additional information in our Zenodo database (doi: 10.5281/zenodo.7813566). This includes all data regarding their optimal intermolecular and lattice energies, the geometric deviations between the optimized DFT-D structures and experimental structures, and also the .res files for the DFT-D optimized crystal structures.

### 4.2 Approach to Parameter Estimation

#### 4.2.1 Selecting a Fitting Methodology

The parameter estimation algorithm can be applied in a number of ways to develop a parameter set. For instance, the choice of atom types based on the local environment, the use of combining rules, or weighting strategies are factors in the fitting of the model. Each of these choices will influence the quality of the fit as well as the transferability of the resulting parameter estimates. Below, we discuss some of the more fundamental choices related to the physical description of the force-field, while decisions related to the numerical settings can be found in the Supporting Information.

First, the choice of which force-field parameters to optimize can strongly influence the goodness-of-fit. The Buckingham potential contains three parameters for each interaction as shown in eq 4. A commonly held view is that accurate determination of the *B* parameter through empirical fitting schemes is challenging and may result in overfitting of the potential. This is usually attributed to its strong correlation with the *A* parameter, as well as the sensitivity of the potential to the *B* parameter value. Following the lead from previous methodologies,^33,34^ we choose to fix the *B* parameter of each potential at the values used in the FIT potential, and only estimate the *A* and *C* parameters for each species–species interaction. It has also been found in previous investigations^33,34^ that explicitly fitting cross-interactions can lead to better models compared to using combining rules. We choose to follow the same methodology and estimate all cross-interactions explicitly.

A second choice related to the physical description of the force-field is the atom types used. Atom types are usually kept as general as possible but a common distinction made is to treat hydrogen atoms connected to a polarizing element such O or N with different parameter values.^8,28−32^ To avoid overfitting, we treat hydrogen atoms bonded to O or N atoms equivalently and denote the atom type as Hp (hydrogen-polarized), whereas hydrogen atoms bonded to carbon or sulfur are denoted Hc.^54^ Considering that hydrogen bonding interactions should be almost entirely stabilized through electrostatic and induction effects, which are not explicitly treated in the empirical component of the potential, the *C* parameters of the Buckingham potential are fixed to zero for all interactions involving Hp. This assumption has also been applied by previous authors.^9,33^ Although some of the AE datasets contain thiol structures with hydrogen bonded to sulfur, these datasets have been omitted from the initial testing done in this work, and thus no sulfur-bonded hydrogen is defined. Whether the sulfur-bonded and carbon-bonded hydrogens *need* to be distinguished is debatable and beyond the scope of this paper.

To maximize transferability of the parameters across systems, it is usually beneficial to fit all parameters in the model simultaneously to prevent error propogation. This ensures, for instance, that the parameters for carbon–carbon interactions are well-conditioned for use beyond simple hydrocarbon systems, such as in the structures of azo-organic or oxy-organic compounds. This has not been done by previous researchers in this area most likely due to the inherent computational complexity. Our implementation is suited for such large-scale problems and optimization of all model parameters simultaneously can be performed more easily. This drastically reduces the number of separate parameter estimations that need to be performed to obtain a full parameter set. While it is hoped that this approach will improve the quality of the resultant parameter estimates, there are cases where it might do otherwise. In the HAIEFF model employed in this work, the effects of induction have not been considered. Without this key contributor to hydrogen bonding effects, force-field parameters that should be unrelated to hydrogen bonding may be wrongly used to compensate for the omitted energy term. This could ultimately hurt the transferability of the final parameter sets. Careful selection of the weighting factors in eq 11 can aid in abating this problem. Since our primary objective in this paper is to test the parameter estimation algorithm, rather than to generate a generic parameter set, the energy weightings for structures containing O–H or N–H bonds are set to zero (i.e., ), while the geometry weightings are fixed at unity (i.e., ). In this manner, energy residuals for structures that can form hydrogen bonds are excluded from the parameter fitting and there is less risk of the previously described error propagation. For all other structures, we select an unbiased weighting scheme (i.e., ) as applied by other authors.^7,34^ Bowskill has shown by *k*-fold validation that the force-fields generated with such a weighting scheme exhibit only minor deterioration in accuracy when used to predict crystal geometries and energies beyond the training data, further justifying this selection.^11^

#### 4.2.2 Selection of Test Problems

When testing the optimization algorithm it is important to understand how the performance of the algorithm is affected by problem size. The largest problems investigated in similar studies include parameter estimations involving 5 structures and 12 parameters in the work of Bowskill et al.,^7^ 19 structures and 6 parameters or 15 structures and 8 parameters in the work of Gatsiou et al.,^34^ and 24 structures and 1 parameter in the work of Pyzer-Knapp et al.^33^ For our purposes, we wish to examine how well our methodology performs on problems of this size as well as much larger estimation problems that have not been tackled by other approaches. To synthesize test problems of various sizes, we opt to use different combinations of the reference datasets from Table 1. The seven test problems we investigate are given in Table 4.

**Table 4:** Seven Test Problems Used to Evaluate the Performance of the Optimization Algorithm

Test Problem	Reference Datasets	Atom Types Present	Number of Parameters	Number of Structures	Number of Residuals
TP1	RS1	C, H	6	18	1,380
TP2	RS1–RS2	C, H, N	12	48	3,009
TP3	RS1–RS3,RS7	C, H, N, O	20	96	6,142
TP4	RS1–RS3,RS5,RS7, RS10–RS11,RS17	C, H, N, O, F	30	140	9,016
TP5	RS1–RS5,RS7–RS12, RS16–RS19,RS23	C, H, N, O, F, Cl	42	229	14,549
TP6	RS1–RS23	C, H, N, O, F, Cl, S	54	313	20,970
TP7	RS1–RS34	C, H, N, O, F, Cl, S, Hp	62	445	28,465

The first test problem (TP1) is typical of the size of problem investigated by other authors. This involves fitting 6 model parameters for the set of interactions between carbon and hydrogen. Only one reference dataset is included in this fitting, containing 18 structures and 1,380 residuals. Thereafter, the test problems grow in size as more species, containing a wider range of atom types, are added to the training. With each additional atom type, the number of fitting parameters increases nonlinearly since cross-interactions are also parametrized. TP2 is already larger than most problems that have been investigated in the literature, both in terms of the number of parameters that are simultaneously estimated and the number of training structures/data points. The largest test problem is TP7, which includes all of the primary reference datasets developed in this work and involves fitting 62 model parameters simultaneously. In this final problem, all 445 structures are included in the fitting resulting in 28,465 residuals. It is important to note that each test problem is solved as an independent parameter estimation, without carrying forward any parameters obtained from preceding tests.

## 5 Results

### 5.1 Analysis of the Reference Dataset

#### 5.1.1 Chemical Profile of the Reference Data

The combined primary datasets and atom environment datasets yield 755 unique DFT-D resolved crystal structures with a total of 46,315 residuals. In the area of inorganic crystal structures, several large-scale databases are being developed through the similar use of solid-state DFT to resolve crystal structures, for instance the Open Quantum Materials Database.^67,68^ As previously mentioned, a similar effort has been mostly absent in the area of organic crystal structures. Other than relatively small DFT-D optimized test sets (<60 structures),^46,51^ the largest collection of published DFT-D optimized organic crystal structures is likely from the work of van de Streek and Neumann^69,70^ wherein they resolved a total of 440 crystal structures. However, usage of their database is complicated by the fact that they utilized different DFT functionals in the two publications (PW-91 in the first^69^ and PBE in the second.^70^ Additionally, since the focus of their work was on validating the use of DFT-D for resolving crystal geometries, only the relaxed crystal geometries were reported without their corresponding optimized energies. In their study of polymorphism, Cruz-Cabeza et al. also conducted large-scale DFT-D optimization on 446 organic crystals,^71^ but the results of their calculations were not published as a database. As such, to our knowledge, our work constitutes the largest repository of self-consistent, high-quality, DFT-D optimized organic crystal structures that includes both the crystal energies and geometries.

**Figure 2:**
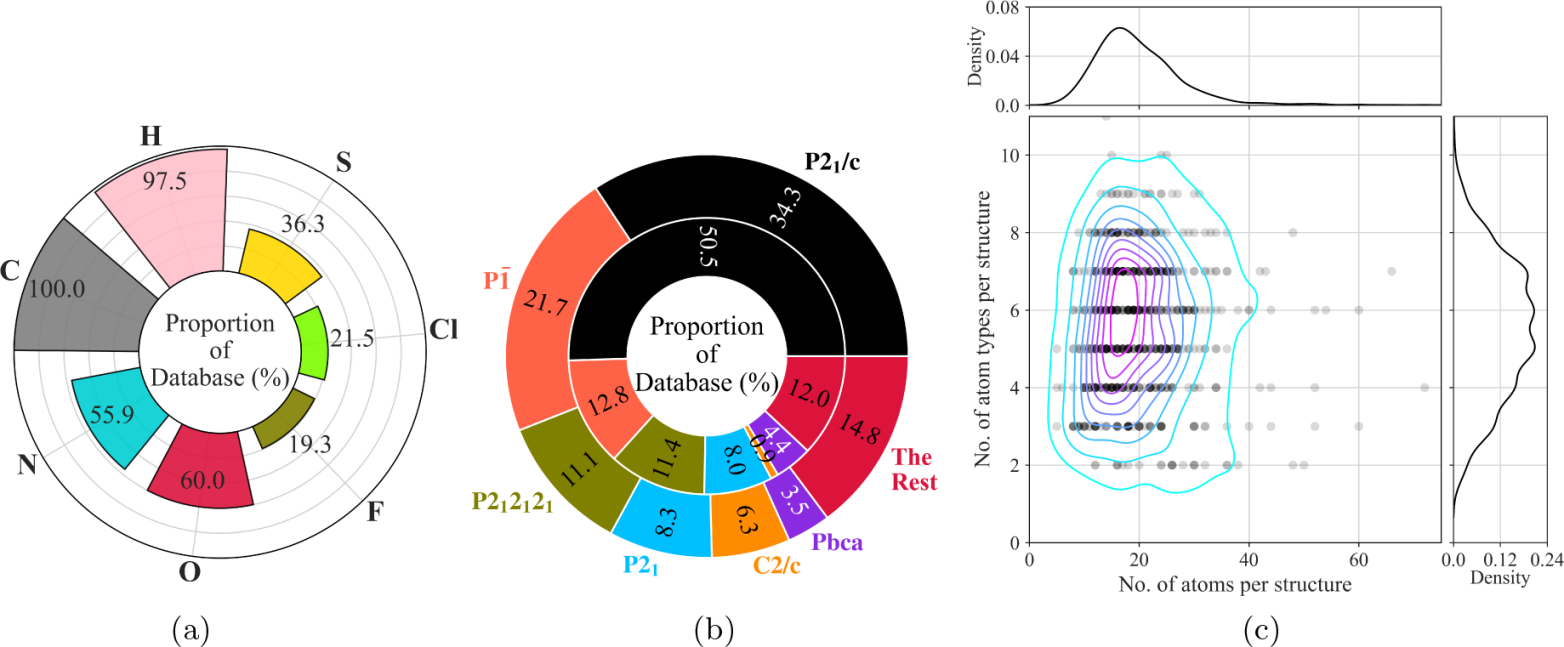
(a) The proportion of structures in our database containing each element. (b) The proportion of structures in our database (inner ring) contained within the 6 most frequently observed space groups for organic crystals, as determined using the CCDC Python API (outer ring). (c) The distribution of structures based on the number of atoms and number of atom types in each structure. The opacity of the black markers reflects the frequency of structures with a given number of atoms and atom types; this is also reflected by the contour lines and density curves. The atom types are as defined in Table 2.

One can expect that in the parametrization of a force-field, transferability would be tied to the breadth of the reference data. In this regard, we consider some general statistics of the full dataset in Figure 2. In terms of the elemental makeup of our database, since all of the chosen crystal structures are organic, all molecules contain carbon atoms and almost all contain hydrogen atoms (polarized or otherwise). Conversely, halogens and sulfurs are relatively scarce within the dataset, but this is to be expected. To assess the space group distribution within our database, we utilize the CCDC’s Python API to determine the relevant statistics by surveying the CSD. In contrast to the statistics reported by the CCDC,^72^ our statistics reflect the distribution for solely organic crystals (as flagged by the Python API). Out of the 1,304,168 entries recorded in the CSD at the time of assessment, only 585,654 entries (about 44.9%) were determined to be organic crystals with valid space groups (this includes repeated entries describing the same polymorphic forms). Although our database correctly reflects  as the most frequently observed space group, it is evident that the composition of space groups in our database does not match well with the composition determined from the CSD. In particular the  should be the fifth most frequently observed space group, yet in our database it has little to no representation. This is primarily because many structures originally in the  space group had fractional *Z*^′^ number. Unfortunately, in its current implementation, CSO-RM (and by extension CrystalEstimator) can only accommodate integer *Z*^′^ values. As such, it was necessary to reduce the symmetry of these structures to achieve the required *Z*^′^. Finally, by nature of our selection process, the database has a clear bias toward small molecules (with <20 atoms), and most of the selected compounds have between 5–7 distinct atomic environments (as defined in Table 2). The full dataset contains a total of 22 polymorphic pairs and 3 polymorphic triplets.

**Figure 3:**
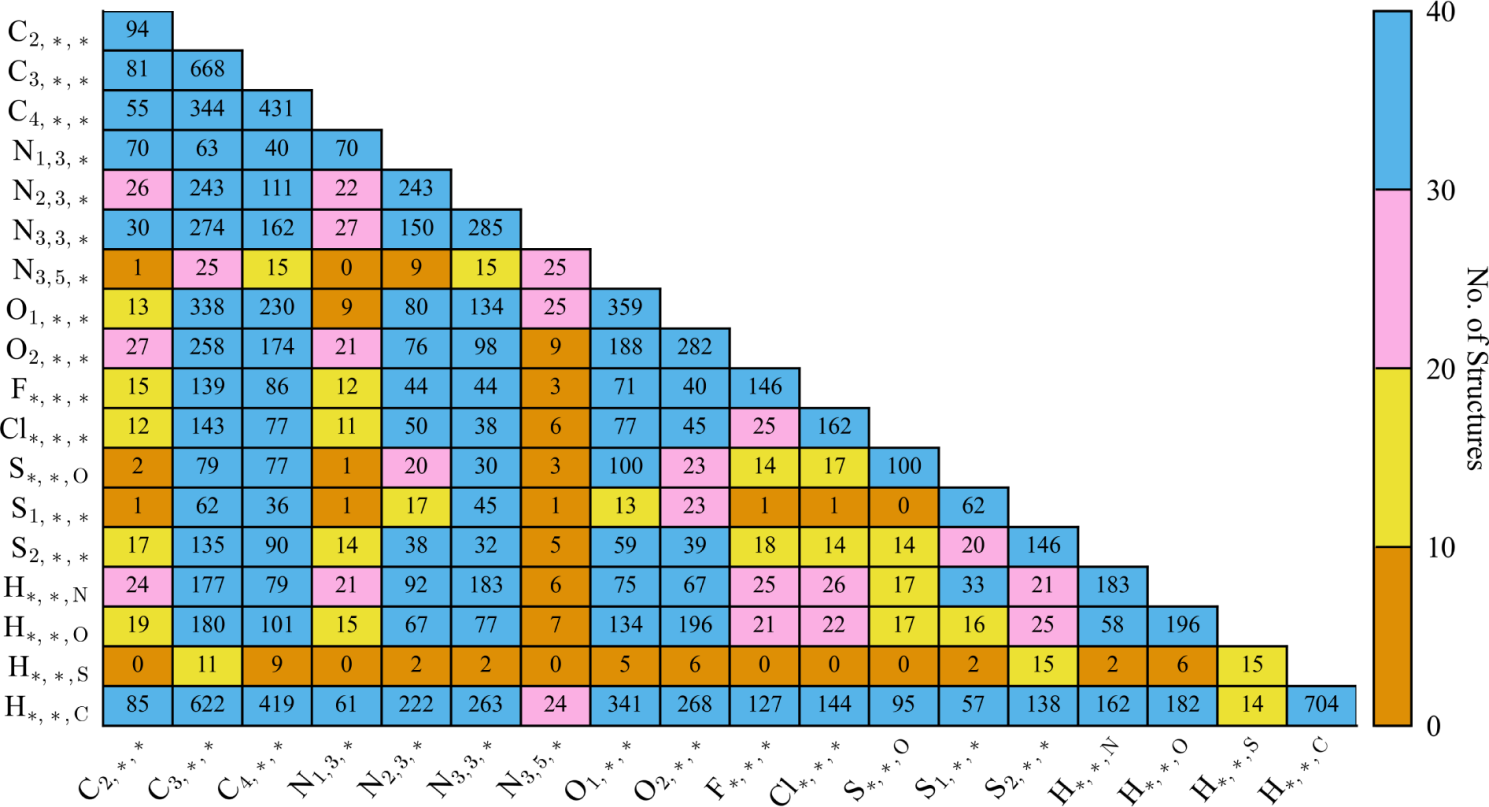
Tally of structures containing each pairwise atomic-interaction within the combined reference dataset. Each element of the matrix corresponds to the total number of structures which contain the pair of atoms on the row and column. Cells are colored orange (<10), yellow ( and ), pink ( and ), and blue () according to the structure counts.

To further ensure that our reference dataset is sufficiently broad, we consider the prevalence of pairwise data between the various environments, as shown in Figure 3. Across the entire matrix, the majority of pairwise interactions are represented by at least 10 structures. While this might seem like a relatively small number of structures, because each structure contains information regarding both the crystal energy and crystal geometry (cell geometry and atomic positions), each structure contributes multiple data points into the parameter fitting objective function. Below, we attempt to rationalize why the remaining interactions do not meet this threshold and the significance of this relative data paucity.

The rows and columns related to N_3,5,*_ and H_*,*,S_ both present with a systematic sparsity of data. These atomic environments are associated with chemical groups that do not hold broad industrial and scientific interest. Consequently, their presence in the CSD, and by extension our database, is proportionately small. N_3,5,*_ is present only in nitro-containing compounds which are primarily used as explosives.^73,74^ In these energetic materials, any presence of nitrogen and oxygen is predominantly from the nitro-groups, while halogens and sulfurs are almost never featured. The spread of data related to the N_3,5,*_ pairwise interactions directly mirrors this. Similarly, H_*,*,S_ is present only in thiol-containing structures. While thiol-compounds are used in several drugs, most commonly as a chelating agent, their presence in pharmaceuticals is relatively niche.^75^ Moreover, most industrially relevant thiol-compounds exhibit significant flexibility (branched, aliphatic compounds with many consecutive sp^3^ carbons). Our attempts to screen the CSD for these structures corroborates this, with the majority of our search results being highly flexible, aliphatic structures ill-suited for our purposes. Aside from these drugs, there does not appear to be much use for organic thiol-compounds in the solid state. Beyond these systematic gaps in the data, there are some isolated groups of missing data. As mentioned before, searches involving S_1,*,*_ with halogens or S_*,*,O_ were unsuccessful at generating a sufficient number of suitable candidates. Likewise, searches involving S_1,*,*_ and S_*,*,O_ paired with other uncommon chemical groups like cyano-groups (C_2,*,*_ + N_1,3,*_) and alkyne-groups (C_2,*,*_ + C_2,*,*_) were also ineffective.

In summary, the regions of missing data are mostly due to a lack of industrially relevant structures containing those pairwise interactions, and to a lesser extent to the limits of flexibility we impose on the datasets. If our goal is to parametrize *useful* CSP force-fields, then it can be reasoned that a lack of data in these “industrially uninteresting” regions should not be a major cause for concern. In the event where it might become necessary to parametrize a force-field with these specific interactions, our use of DFT-D liberates us from the need to rely on experimentally observed structure, and offers the flexibility to generate hypothetical structures containing the interactions of interest. This is akin to what is already practiced in the parametrization of TMFFs.

Another facet of the diversity in the dataset that is not captured by the pairwise interaction matrix is the presence of different hydrogen bond motifs. As noted in ref (11), it is the description of these hydrogen bonding structures which tends to pose the biggest challenge to the force-field parametrization. During the database construction, we identified potential intermolecular hydrogen bonds solely from the presence of H_*,*,O_ or H_*,*,N_ atoms in the molecule, however this approach is inadequate for the intended analysis here. In some structures, there are multiple valid donor/acceptor pairings and the actual hydrogen bonding motifs cannot be inferred from the molecular description alone. Also, several molecules have conformations that could favor intramolecular hydrogen bonds over intermolecular hydrogen bonds. In reality, even among polymorphs of the same molecule, hydrogen bonding motifs can differ,^71^ further emphasizing the need for a more robust approach to elucidate the presence of intermolecular hydrogen bonds within the crystals. To that end, we employ the CCDC’s Python API to identify hydrogen bonds through geometric criteria (details in the Supporting Information). The effect of applying these criteria is immediately apparent. Analysis of the primary datasets based solely on the presence of H_*,*,O_ and/or H_*,*,N_ suggests that they contain 132 hydrogen bonding structures. Conversely, the geometric approach identifies only 126 structures containing intermolecular hydrogen bonds. In the full dataset, using the geometric criteria, 314 hydrogen bonding structures are found, approximately 41.6% of the entire dataset. A more detailed breakdown of the motifs present within these 314 structures is summarized in Table 5. While hydrogen bonding motifs, as defined using graph sets,^76,77^ have a more formal definition that also considers the nature of the repeated hydrogen bonding pattern, in the interest of simplicity, we only consider the identity of the donor and acceptor atoms.

**Table 5:** Number of Occurrences of Each Hydrogen Bonding Motif within the Combined Reference Dataset^a^

	X = N	X = O	X = S	Total
Y = N	144	84	2	230
Y = O	128	165	5	298
Y = S	49	14	15	78
Y = Cl	10	4	-	14
Total	331	267	22	620

a Each value corresponds to the total number of X–H···Y motifs identified across all structures. A single motif can occur multiple times in a given structure.

Naturally, given their greater presence in the CSD, motifs involving a N–H or O–H hydrogen bonded to N or O form the majority of the motifs in our dataset. This could also be a reflection of the fact that hydrogen bonds involving oxygen and nitrogen tend to be “stronger” and are more stabilizing due to the greater polarizing effect exerted by the period 2 elements, and as such the formation of these hydrogen bonds is more favorable. It is also encouraging to observe that noncanonical hydrogen bonds involving the heavier elements, like sulfur and chlorine, are not completely absent from our dataset, although they are relative scarce. Ultimately, the analysis of the atomic environments and hydrogen bonding motifs indicates that our reference dataset has a sufficiently broad chemical profile for use in parameter estimation of transferable force-fields. Areas in the dataset where information appears sparse can mostly be attributed to the scarcity of relevant structures in the CSD.

#### 5.1.2 Validation of Reference Data

While the breadth of our reference data appears sufficient, it is also crucial to assess the accuracy and reliability of the reference datasets. To begin, we first validate our results against similar calculations performed by Moellmann and Grimme^50^ on the X23 test set^37^ using a number of different functionals, including the TPSS functional with the D3 dispersion correction. Of the 23 structures analyzed in their study, 12 structures also appear in our reference datasets and can be directly compared to assess the quality of the DFT-D calculations performed in this work. The computed lattice energies for the X23 test set, DFT-D energies from the work of Moellmann and Grimme and the DFT-D energies in this work are shown in Table 6, showing very good agreement in the DFT-D calculations for most structures, although slightly larger deviations are observed for acetic acid and succinic acid, both of which contain hydrogen bonds in their crystal structures. These deviations may be attributed to slight differences in the numerical settings used, such as the density of *k*-point sampling, as well as the use of different versions of the VASP software. Compared to the X23 test set, the average deviations are calculated to be rather small, at 0.2 kJ/mol in both cases. The average absolute deviations and standard deviations are also small and below chemical accuracy of 4.2 kJ/mol. Slightly better results are obtained in our work when compared to the X23 test set, with an absolute average deviation and an unbiased standard deviation of 2.5 and 3.7 kJ/mol, respectively.

**Table 6:** Comparison of Lattice Energies from the X23 Test Set,^37^ TPSS+D3 Calculations Produced by Moellmann and Grimme,^50^ and TPSS+D3 Calculations Performed in this Work^a^

	Predicted Lattice Energies (kJ/mol)		
Compound	X23^37^	Moellmann and Grimme^50^ (TPSS+D3)	This Work (TPSS+D3)
1,4-cyclohexanedione	–88.6	–87.4	–86.8
Adamantane	–69.4	–69.9	–69.3
Anthracene	–112.7	–114.2	–113.0
Benzene	–51.7	–55.6	–55.0
Hexamine	–86.2	–86.6	–86.2
Naphthalene	–81.7	–84.5	–83.9
Pyrazine	–61.3	–66.1	–65.3
Triazine	–61.7	–60.7	–60.4
Trioxane	–66.4	–58.2	–58.0
Acetic acid	–72.8	–68.2	–73.1
Imidazole	–86.8	–91.2	–91.1
Succinic acid	–130.3	–129.3	–132.4
AD (kJ/mol)	-	0.2	0.2
AAD (kJ/mol)	-	2.9	2.5
SD (kJ/mol)	-	3.8	3.7

a Average deviations (AD), absolute average deviations (AAD), and unbiased standard deviations (SD) are calculated relative to the X23 test set lattice energies.

Next, we wish to compare the results obtained in this work against experimental data. First, the average geometry deviations between the experimental and DFT-D optimized structures are measured by evaluating the average RMSD_15_ over all 755 structures in the combined reference datasets. This is done using the CCDC’s COMPACK tool,^66^ keeping all calculation settings at their default values. On average, both the non-hydrogen bonding and hydrogen bonding structures are reproduced well by theoretical calculations, with average RMSD_15_ values of 0.1068 and 0.1127 Å respectively. Given the effects of finite-temperature on crystal geometry, the geometry predictions of the reference datasets seem to be in line with the experimental data.

To validate the accuracy of the calculated lattice energies, we compare the DFT-D determined lattice energies against experimental sublimation enthalpy data. The experimental data used here were curated by Gatsiou and coworkers^34^ and Marchese Robinson et al.^78^ In the latter source, some of the structures are quoted with different CSD Refcodes relative to the structures in our database. An RMSD_50_ comparison is done between the two CSD Refcodes to validate that each experimental value quoted is indeed in relation to the same structure within our database. We do not face the same ambiguity in the former source since the structures in our primary datasets contain all the structures from ref (34) with the same Refcodes. To convert the finite-temperature sublimation enthalpies into static, 0 K lattice energies, the Dulong-Petit law is used as an approximate correction for temperature effects.^37,38^ Between the two databases, there can be multiple reports of sublimation enthalpies for the same structure. There is no objective criterion to judge the reliability of each individual source, hence we simply consider the range of the reported data. The results of this comparison are summarized in Tables 7 and 8. A full list of all the experimental data used can be found in Table S1.

**Table 7:** Comparison of Experimentally-Derived Lattice Energies () with the DFT-D Computed Lattice Energies () for Non-Hydrogen Bonding Structures^a^

REFCODE	(kJ/mol)	#	(kJ/mol)	Δ*U*_*latt*_ (kJ/mol)	Source(s)
ADAMAN08	–69.28	1	–63.96	5.32	(79)
ANTCEN16	–113.00	1	–102.86	10.14	(80)
BENZEN06	–55.02	1	–49.36	5.66	(81)
BIPYRL04	–96.88	3	[−86.86,−79.96]	[10.03,16.93]	(82−84)
BNZQUI03	–76.24	4	[−71.66,−67.72]	[4.58,8.52]	(85−87)
BZPHAN01	–124.38	1	–110.96	13.42	(88)
CLMETH03	–33.15	1	–34.11	–0.96	(89)
CRYSEN	–138.77	1	–128.36	10.41	(90)
CUBANE	–56.48	1	–60.16	–3.68	(91)
CUKCIU03	–54.38	1	–40.12	14.26	(92)
CYHEXO	–86.79	1	–79.96	6.83	(93)
DCLANT10	–123.74	1	–119.65	4.09	(94)
DCLBQN	–82.73	1	–74.86	7.88	(87)
DITHAN02	–84.60	3	[−73.86,−67.96]	[10.75,16.65]	(95,96)
DMSULO04	–83.79	1	–81.96	1.83	(97)
DNBENZ11	–85.32	1	–91.96	–6.63	(98)
DTENYL02	–96.43	1	–90.16	6.27	(99)
FABPON11	–138.61	1	–128.26	10.35	(100)
HCLBNZ13	–100.14	1	–95.46	4.69	(94)
HEXANE01	–51.67	1	–53.76	–2.09	(101)
HXMTAM10	–85.17	2	[−84.56,−79.86]	[0.61,5.31]	(102,103)
NAPHTA31	–83.65	14	[−78.16,−68.96]	[5.49,14.69]	(90,96,104−115)
OCTANE10	–67.34	1	–71.69	–4.35	(101)
PENTAN01	–43.33	1	–44.38	–1.05	(101)
PERLEN06	–148.57	1	–136.50	12.07	(116)
PHENAN08	–106.09	1	–95.86	10.23	(112)
PHENAZ04	–106.40	1	–100.19	6.22	(117)
PNCLBZ	–93.14	1	–91.98	1.16	(78)
PRMDIN01	–65.09	1	–63.26	1.83	(78)
PTOXEC	–76.73	2	[−92.86,−92.82]	[−16.12,−16.09]	(78,118)
PYRAZI01	–65.26	1	–61.16	4.10	(119)
PYRENE03	–113.74	1	–105.26	8.49	(90)
SUCANH12	–78.54	2	–85.66	–7.12	(78,120)
TCYETY01	–85.42	1	–86.16	–0.73	(121)
TETDAM03	–70.45	1	–67.16	3.29	(122)
TOXOCN	–72.01	2	[−84.56,−84.45]	[−12.55,–12.44]	(78,123)
TRIPHE13	–133.93	1	–125.06	8.88	(90)
TRITAN03	–109.71	1	–98.16	11.56	(124)
TRIZIN01	–59.88	4	[−61.85,−59.14]	[−1.97,0.74]	(96,125)
TROXAN	–56.98	1	–61.56	–4.58	(126)

a The number of measurements associated with each structure is given (#), and if multiple sources give differing experimental values, both  and the deviations in *U*_*latt*_ () are instead reported as ranges. Positive values of Δ*U*_*latt*_ indicate overbinding.

**Table 8:** Comparison of Experimentally-Derived Lattice Energies () with the DFT-D Computed Lattice Energies () for Hydrogen Bonding Structures^a^

REFCODE	(kJ/mol)	#	(kJ/mol)	Δ*U*_*latt*_ (kJ/mol)	Source(s)
ACETAC07	–73.15	1	–70.68	2.46	(127)
AMBNAC12	–125.91	2	–121.06	4.85	(128,129)
AMPYRE	–104.43	3	[−93.06,−92.06]	[11.37,12.37]	(130,131)
AMPYRM11	–96.08	1	–81.46	14.62	(130)
BAGFIY01	–102.83	1	–90.46	12.37	(132)
BENZAC12	–102.67	1	–94.96	7.71	(133)
BESKAL04	–127.39	1	–135.36	–7.96	(134)
BZDMAZ02	–111.69	5	[−111.96,−99.26]	[−0.27,12.43]	(96),^135−137^
CLBZAC01	–116.31	2	[−111.26,−105.86]	[5.05,10.45]	(138,139)
FORMAC02	–67.58	1	–65.06	2.51	(79)
FULPIM	–96.27	1	–93.36	2.91	(140)
HXQUIN14	–91.43	5	[−113.96,−92.86]	[−22.52,−1.42]	(141−143)
IMAZOL06	–91.12	1	–86.36	4.76	(135)
MALIAC12	–117.02	1	–110.36	6.66	(144)
MELAMI06	–150.94	2	[−130.48,−124.96]	[20.46,25.98]	(79,145)
NAPOAC01	–127.98	1	–118.56	9.42	(146)
PFBZAC	–92.12	1	–96.46	–4.34	(147)
SALMID04	–115.45	2	[−106.86,−104.26]	[8.59,11.19]	(148,149)
SUCACB03	–132.44	1	–128.06	4.38	(150)
TRAZOL03	–91.28	2	[−89.05,−87.96]	[2.22,3.32]	(78,151)
XAGWIM	–112.43	1	–105.76	6.67	(152)
ZZZKPE03	–103.02	1	–91.6	11.42	(153)

a The number of measurements associated with each structure is given (#), and if multiple sources give differing experimental values, both  and the deviations in *U*_*latt*_ () are instead reported as ranges. Positive values of Δ*U*_*latt*_ indicate overbinding.

On average, the calculations using the TPSS functional and D3 dispersion correction appear to overbind the crystal between 3.90–5.24 kJ/mol compared to the experimentally derived results. This propensity to overbind is marginally greater for hydrogen bonding structures (4.70–6.94 kJ/mol) compared to non-hydrogen bonding structures (3.47–4.30 kJ/mol). The largest deviations in energies are observed for the crystal structure of melamine (MELAMI06) and quinolin-8-ol (HXQUIN14), both of which are hydrogen bonding. For MELAMI06, the two available experimental data points suggest that DFT-D overbinds the crystal by 20.46 kJ/mol^79^ and 25.98 kJ/mol.^145^ The sublimation enthalpy data obtained from ref (79) was determined at an elevated temperature of 432 K, which one could potentially point to as a source of large errors when the temperature correction is applied. However, the enthalpy data obtained from ref (145) was determined at 298.15 K, making the temperature-correction argument less valid at explaining the calculated overbinding. Furthermore, if the reported experimental measurement error for this source ( kJ/mol) is taken into consideration, both sources are actually in good agreement that the DFT-D calculated lattice energy for MELAMI06 may be too negative. For HXQUIN14, there are five different experimental measurements quoted,^141−143^ all obtained at 298.15 K. The five measurements can be split into two groups: two measurements with reported experimental sublimation enthalpies within 0.2 kJ/mol of each other, and three measurements with reported experimental sublimation enthalpies within 1.6 kJ/mol of each other. The average sublimation enthalpies of these two groups differ by 20.1 kJ/mol, which explains the large range in the errors for HXQUIN14. The lower bound on the error-range corresponds to the group of two and the upper bound to the group of three. While both groups of results concur that the DFT-D calculation underbinds HXQUIN14, the extent of this error cannot be conclusively determined based on these two sets of conflicting experimental results.

The unbiased standard deviation of the energy difference across all the experimental data is also large at 8.11 kJ/mol, suggesting significant differences between the experimental and DFT-D results. This standard deviation is more than twice the standard deviation observed in relation to the X23 test set energies reported in Table 6. In our view, the large variability in the energy errors is not an indictment of the accuracy of our DFT-D calculations, but rather a demonstration of the shortcomings of using experimentally determined sublimation energies. As can be seen from Tables 7 and 8, where multiple experimental results are found, there is often significant inconsistency in the reported sublimation enthalpies, which hampers our ability to use these measurements effectively. This is further compounded by the complications involved in carrying out thermal corrections to obtain accurate static, lattice energies. For instance, the sublimation enthalpy values and thermal corrections used in the work of Gatsiou and coworkers are often at odds with the lattice energies determined in the X23 test set: the lattice energies of adamantane and anthracene crystals used in the work of Gatsiou and coworkers and the X23 test set differ by 5.4 and 9.8 kJ/mol, respectively, despite both methods deriving their results from sublimation enthalpy data. Altogether, this highlights the continuing challenge of obtaining accurate lattice energies from sublimation enthalpy data, further supporting our decision to employ DFT-D generated data instead.

**Figure 4:**
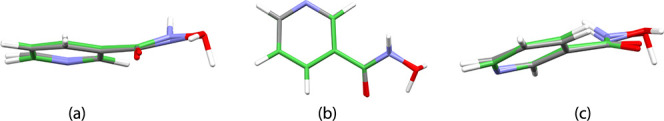
RMSD_1_ overlay of the FUZZAB molecule in its equilibrium conformation (gray) and crystalline-phase conformation (green), viewed from (a) the side of the aromatic ring’s plane, (b) orthogonal to the aromatic ring’s “face”, and (c) along the axis of the C–N bond. In the equilibrium conformation, the O–H is almost planar with the carbonyl oxygen while in the crystalline-phase, the O–H orientates orthogonally to the carbonyl oxygen.

The intermolecular energy used as reference data in the parameter estimation is determined by removal of the intramolecular cost of deforming the molecule from its *in vacuo* conformation from the overall lattice energy. As structures are preferentially selected to include mostly rigid molecules, we expect the intramolecular contributions to lattice energy to be small for most structures. For the non-hydrogen bonding structures in our reference datasets, the intramolecular component of the lattice energy is predicted to be 1.77 kJ/mol on average, within the range of 0.08–9.94 kJ/mol. In these structures, the differences in intramolecular energy usually correspond to small changes in the equilibrium geometry of the molecule as the molecular structure is strained by intermolecular packing forces. In other cases, large intramolecular contributions can be due to the chosen molecules exhibiting varying degrees of flexibility. While this contradicts our intention of using rigid molecules, it is sometimes unavoidable since certain atomic environments are inherently found in flexible molecules. On the other hand, the average intramolecular energy for the hydrogen bonding structures is much larger at 10.79 kJ/mol, with a range of 0.50–38.90 kJ/mol. Like the non-hydrogen bonding structures, most of the hydrogen bonding molecules do not exhibit significant changes between the crystalline environment and the gas phase. Despite this, a large intramolecular energy penalty is still incurred, suggesting that the observed molecular adjustments are highly destabilizing and are only tenable in the context of promoting intermolecular hydrogen bond formation. For example, acetic acid has a predicted intramolecular energy contribution of 12.0 kJ/mol despite very small differences in the conformation of the molecule between its optimal gaseous and crystalline state, as reflected by the small RMSD_1_ value of 0.018 Å. The largest change is for the hydrogen atom position in the O–H bond. In the crystalline state, the O–H bond length is extended by approximately 5% from 0.96 to 1.03 Å with a slight increase in the C–O–H bond angle. This perturbation leads to large energetic costs when not surrounded by the highly polarizing environment of a hydrogen bond, but in the crystal it allows the acetic acid molecule to take advantage of intermolecular hydrogen bonds with its neighbors. In more extreme cases, the conformational adjustment can even be at the expense of an existing intramolecular hydrogen bond. For instance, nicotinohydroxamic acid (FUZZAB) has the largest predicted intramolecular energy contribution (38.90 kJ/mol). If only the heavy elements of the molecule are considered, the RMSD_1_ between the equilibrium and crystal phase conformation is 0.098 Å, which when the two conformations are overlaid is rather minor (Figure 4). In the equilibrium conformation, the hydrogen connected to the oxygen in the acid group is orientated in the same plane as the nearby carbonyl oxygen, facilitating strong intramolecular hydrogen bond formation. Conversely, in the crystalline conformation, this planarity is broken as the O–H bond is orientated almost orthogonally to the carbonyl oxygen. This conformational change allows the hydrogen to form hydrogen bonds with a neighboring pyridine group, but at the cost of a large intramolecular penalty. It therefore seems that the rigid-body assumption breaks down for systems that contain hydrogen bonding, and that the flexibility of the polarized hydrogen should be taken into account to model these systems accurately.

Overall, we consider the accuracy of the DFT-D calculations to be sufficient to determine reliable parameter estimates for the intermolecular potential of our hybrid models. The agreement between our lattice energy calculations and the X23 test set and the work of Moellmann and Grimme is encouraging, as is the high accuracy of the geometry predictions across the entire reference datasets relative to experimentally resolved structures. The energies obtained differ much more significantly when compared to experimental sublimation enthalpies, but the reliability of the experimental values are uncertain given that significant disagreements can be observed between the various sources. We find that intramolecular contributions for non-hydrogen bonding structures are generally quite small, but such contributions are much larger for structures that involve hydrogen bonds.

### 5.2 Testing the Performance of the Parameter Estimation Algorithm

#### 5.2.1 Convergence of the Optimization

Applying our implementation of the parameter estimation approach, we can evaluate the behavior of algorithm as the problem size grows. For the purposes of testing, we initially perform the parameter estimation for a simple model based on point-charges. As per Figure 1, the result of each local optimization can be categorized into 1) runs that converged to an optimum and for which the *TMF* is unchanged upon re-evaluation (i.e., satisfies B3 in Figure 1), 2) runs that did not converge to an optimum but terminated due to a slow decrease or increase in the *TMF* (i.e., a suboptimal point that satisfies B4 or B5 in Figure 1), and 3) runs that terminated prematurely due to a failure at some stage of the algorithm. None of the runs terminated due to reaching the specified limit on the number of reinitialisations (B6 in Figure 1). It is also interesting to analyze the number of unique minima that are found in the parameter space by examining the results obtained across all of the Sobol’ seeds.

The results for each of the test problems using a point-charge electrostatic model are summarized in Table 9. For the smaller test problems (TP1–TP4) the algorithm performs remarkably well. The number of Sobol’ points from which the algorithm converges to a locally minimum point is very high, with no optimization failures, and only a few suboptimal points identified in TP1 and TP3. Reasons for nonconvergence in these cases can be attributed to small discontinuities in the *TMF* close to the optimal solution of the parameter estimation problem. Even then, the suboptimal points in TP1 and TP3 achieve objective function values within <1% of the lowest minimum found by the remaining optimizations.

**Table 9:** Overview of Optimization Results for All Seven Test Problems from 128 Separate Sobol’ Points^a^

Test Problem	Runs terminated by criteria B3	Runs terminated by criteria B4 or B5	Failed optimizations	Unique minima
TP1	127	1	0	1
TP2	128	0	0	2
TP3	115	13	0	1
TP4	128	0	0	2
TP5	0	127	1	0
TP6	78	50	0	1
TP7	0	127	1	0

a The respective termination criteria are as described in Figure 1.

After clustering of the results, between one and two unique minima are identified for TP1 through TP4. Relatively strict criteria are used when clustering the minima: both the objective function value as well as the scaled parameter values must be in agreement between two minima for them to be clustered together. In TP2 and TP4, two distinct minima are reported. In both test problems, the minima correspond to fairly similar objective function values, with a relative difference of only 0.7% and 0.1% respectively. In contrast, the scaled parameter values between the minima differ by as much as 71.9% and 56.7% for TP2 and TP4 respectively. The reason why the parameter values between minima are able to differ so much without significantly influencing the resulting objective function values might stem from the choice to simultaneously optimize *A* and *C* for every pairwise interaction. There is inherently some correlation between these two parameters: greater repulsions due to a larger *A* value can be counterbalanced by greater attractions from a larger *C* value (and vice versa if their values are decreased). This seems to be reflected in the two of minima of these test problems, wherein one minimum contains a set of larger *A* and *C* values while the other contains the contrasting pair of smaller *A* and *C* values. The correlation between *A* and *C* is not exact, hence the inclusion of more data could aid in breaking the close degeneracy in these solutions. Ultimately, the discovery of multiple minima on the landscape highlights the need for global optimization techniques such as Sobol’ sampling to thoroughly explore the parameter space. The high convergence rate and the small number of minima identified suggest that the best solution has been found for each of these test problems within the sampling range of the Sobol’ sequence.

**Figure 5:**
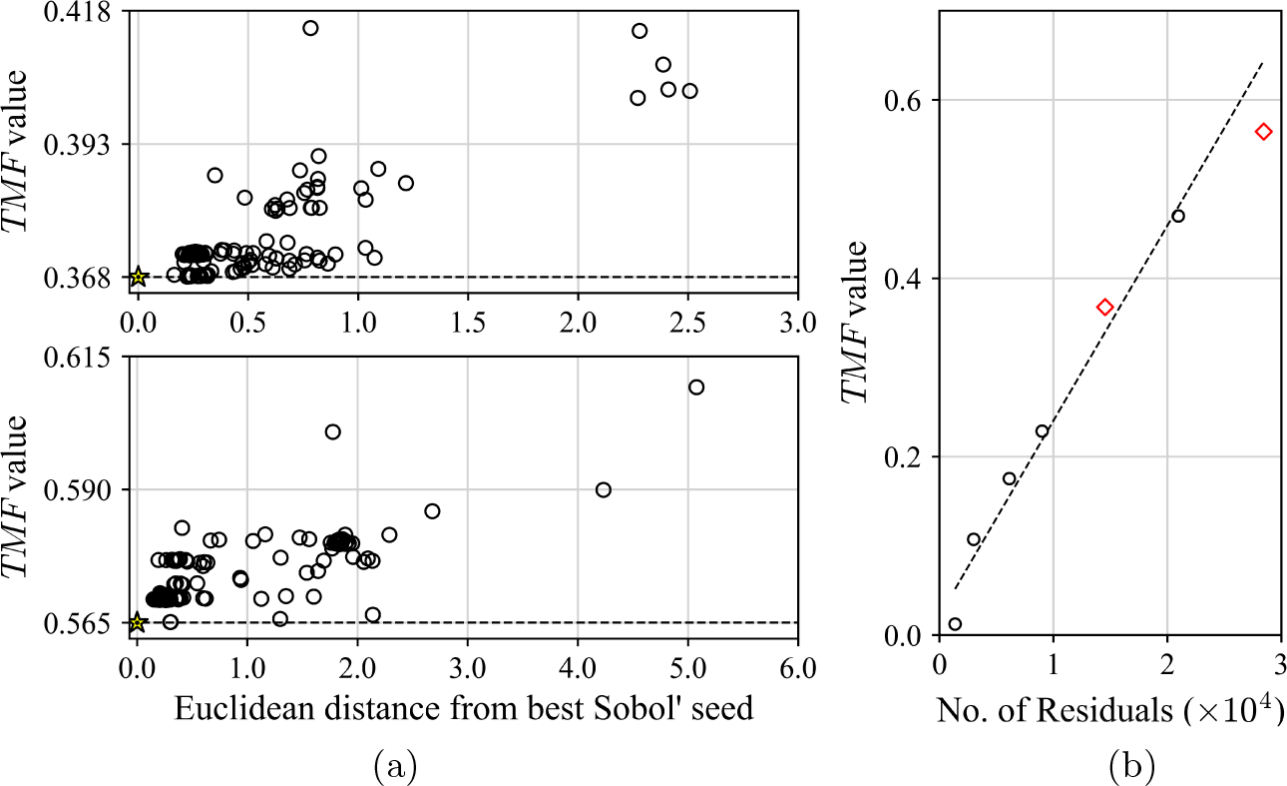
(a) Final value of the *TMF* obtained from CrystalEstimator for TP5 (top) and TP7 (bottom), starting from 127 Sobol’ points (excludes the failed optimization). The best *TMF* value is given by . Distances are in terms of the scaled parameter values. (b) Comparison of the best *TMF* value obtained for different numbers of residuals (i.e., different test problems). Solutions can be either from runs that converged to a locally minimum point (○) or runs that did not (red ◇). The dashed line is the regression line for only the locally optimum solutions (TP1–TP4 and TP6)

As the size of the test problem grows, convergence of the algorithm becomes progressively more challenging. No optimization converged to a locally minimum point for TP5 and TP7, and instead the vast majority of the optimizations terminate based on the alternate termination criteria. A few algorithmic failures are also recorded for these problems, which arise when a significant number of structures fail the lattice energy minimization, indicating that the optimizer has entered a region of unphysical parameter values. Under this circumstance, the optimizer is unable to recover and the algorithm terminates prematurely. For TP6, slightly over half of the optimizations converged to the same locally minimum point while the rest of the optimizations converged to a suboptimal point. Again, the reasons for poor convergence of the algorithm appear to be small discontinuities in the *TMF* at or close to its minimum point(s). Despite this, the parameter estimates obtained from suboptimal convergence can still be adopted. The final *TMF* values obtained from each Sobol’ point are plotted in Figure 5a for TP5 and TP7. For both, although no locally minimum points are identified, most of the optimizations achieve similarly low values for the final *TMF* value. In general, with increasing dissimilarity to the best Sobol’ solution (as indicated by the Euclidean distance), the *TMF* value exhibits a corresponding increase. In Figure 5b, the trend between the number of residuals and the best *TMF* value observed for each test problem is shown. Although TP5 and TP7 do not converge to an optima, their *TMF* values adhere well to the projected trend for the remaining test problems (in fact the best solution in TP7 lies below the trendline). Altogether, these indicate that while the very best possible values parameter estimates may not have been obtained in TP5 and TP7, the algorithm is progressing toward the correct region in solution space and the final *TMF* is likely in the proximity of the true optimum.

#### 5.2.2 Computational Cost of the Optimization

Performance statistics for each of the test problems using the modified local optimization procedure are shown in Table 10. As the number of optimization variables (i.e., parameters) increases across the test problems, so does the average number of total merit function evaluations, as expected.

**Table 10:** Overview of Performance Statistics for All Seven Test Problems Using the Modified Local Optimization Procedure from 128 Separate Sobol’ Points

Test Problem	Average no. of total merit function evaluations	Average no. of lattice minimizations	Average CPU time (hr:min:sec)
TP1	26.2	471.1	00:02:13
TP2	30.0	1,438.9	00:08:07
TP3	32.0	3,075.8	00:20:52
TP4	44.2	6,190.6	00:42:42
TP5	57.4	13,142.5	01:37:55
TP6	53.6	16,777.2	02:21:37
TP7	65.4	29,123.1	03:53:34

CPU times are determined from single core calculations using an Intel Xeon 2.50 GHz CPU. The average CPU time per Sobol’ point correlates almost linearly with the number of structure optimizations. On average, the smallest test problem (TP1) takes approximately 2 min for the termination criteria to be reached from each Sobol’ point, whereas the largest problem takes nearly 4 h. Across the test problems, there is a slight trend of increasing computational cost per lattice minimization, although this may be due to the inclusion of larger molecules in the later reference sets. By fitting a linear correlation between the average number of lattice minimizations and the average CPU time, it is estimated that the algorithm takes 0.48 s per lattice minimization for the reference dataset structures. This includes the time taken for the calculation of the Hessian matrices and residual gradients.

The most similar study of this kind was reported by Bowskill et al.^7^ and involved the fitting of 12 parameters to 5 structures for a point-charge based model. Comparison of the performance statistics in Table 10 with the approximate computational times reported by Bowskill et al.^7^ suggests a computational cost reduction of approximately 2–3 orders of magnitude per Sobol’ point optimization using the new methodology. These major savings in computational cost are primarily the result of using analytical gradients as opposed to numerical ones, faster lattice minimizations using CSO-RM, as well as some of the other optimization strategies discussed earlier. Analytical gradient calculations significantly reduce the computational cost of each iteration relative to finite difference approaches, while the improved accuracy in the gradients may also reduce the number of iterations required for the algorithm to reach termination. The number of Sobol’ samples used for each of the test problems is also significantly reduced (by approximately an order of magnitude) compared to the work of Bowskill et al.,^7^ but the sample size used here seems more than sufficient to achieve reliable parameter estimates. This is likely to be a reflection of the more robust local optimization procedure, which allows the parameter space to be sampled much more sparsely without any loss in the performance of the best parameter estimates obtained. The combination of faster optimizations and reduced sampling leads to a reduction in overall computational cost of 3–4 orders of magnitude.

#### 5.2.3 Parallel Implementation

**Figure 6:**
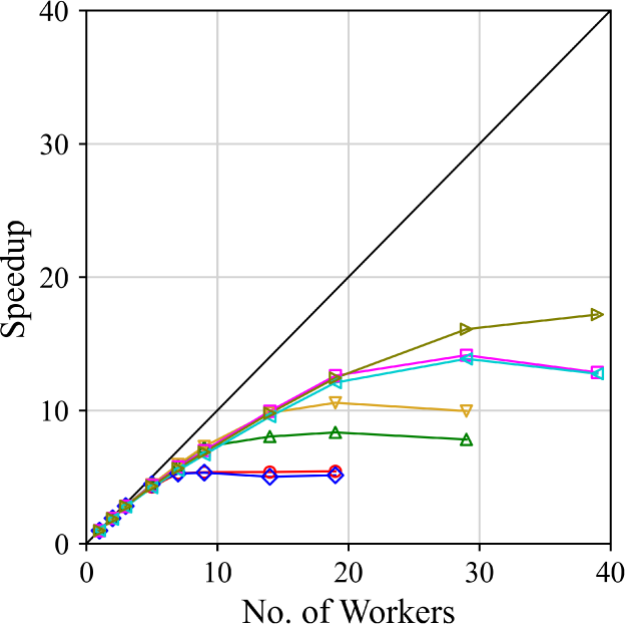
Plot of code speedup for the optimization of a single Sobol’ point for each test problem as a function of the number of workers (). The solid black line represents perfect speedup and the markers are for each test: TP1 (red ○), TP2 (blue ◇), TP3 (green △), TP4 (orange ▽), TP5 (magenta □), TP6 (aqua blue ◁), and TP7 (olive ▷).

All calculations so far have been performed in serial operation without leveraging the parallel implementation of CrystalEstimator. To test the effectiveness of the parallel implementation, one Sobol’ point optimization is run for each of the seven test problems using different core sizes (*N*_*c*_) across a single node. Code speedup relative to serial operation is then analyzed based on the number of workers () used to perform the lattice minimizations and residual calculations. The speedup achieved for each test problem is plotted in Figure 6. Again, all calculations are performed using Intel Xeon 2.50 GHz CPUs.

For a core size of , only one worker is used and ideally the computational time should be equivalent to that obtained in serial operation. However, due to additional core communication time in parallel operation, there is a slight increase in the total computational time of approximately 1–3% using  for all test problems. For low core sizes (), the speedup achieved is relatively close to perfect speedup behavior. For TP1 and TP2, the maximum speedup is achieved at approximately  where larger core sizes fail to provide any real benefit as worker idle time becomes more of a factor. As the size of the test problem increases so does the core size at which the speedup plateaus. This happens between 10–15 cores for TP3, and 15–20 cores for TP4. Additional gains above 20 cores seem to diminish even for the larger TP5, TP6, and TP7 test problems.

While analyzing the factors limiting speedup of the code, it appears that it is not always core communication nor worker idle time that are limiting speedup of the code. Additionally, for large core sizes, the speedup actually starts to fall as core size is increased further. This is most easily observed for TP5 and TP6 but is also present in the other test problems. The additional computational time taken arises from slower lattice minimizations. For TP7, the calculation times for a single lattice minimization increases by approximately 50% on average using 40 cores relative to serial operation. A likely explanation for this is that the introduction of additional cores slows down each of the individual lattice minimizations performed on their respective cores. This could be due to an increased number of load cache misses as multiple MPI processors are running simultaneously across shared-cache memory.^154^ One potential mitigation strategy could be to distribute the same number of cores over multiple nodes. Since each MPI process can access only its own node’s local memory, there should be less competition for physical memory and cache resources. However, this could introduce other bottlenecks in terms of internode communication. A more detailed profiling of the program bottlenecks would be desirable to clearly diagnose the barriers to better scalability.

#### 5.2.4 Optimisation of Multipole Based Models

The convergence behavior of the algorithm when fitting multipole based electrostatic models is also analyzed against the seven test problems of this study. The steps followed by the algorithm are essentially identical to that followed for the point-charge model except that lattice minimizations and second derivative calculations are performed with the inclusion of all multipole interactions up to . Multipole based models are significantly more complex than their point-charge counterparts. The resulting summations involve additional terms that must be calculated for each atom–atom interaction. As a result, the computational cost of performing evaluations of intermolecular energy and its derivatives is approximately an order of magnitude larger for this type of model.

The convergence of the algorithm for all test problems across 128 Sobol’ point optimizations can be found in Table S2. The behavior of the algorithm appears to be broadly equivalent to the point-charge model. A slightly higher proportion of algorithmic failures and convergence to suboptimal solutions is observed, although the difference is not significant. Roughly the same number of unique minima are identified as before, ranging between 1 and 3 minima found per test problem. We also find that convergence to a locally minimum point does not guarantee that the very best solution has been found. In fact, for TP6 and TP7, many of the points that did not converge to a locally minimum point achieve lower objective function values than any of the local minima identified.

The number of total merit function evaluations to reach convergence is approximately the same as for the point-charge model. Accurate determination of the relative computational cost of the multipole model is challenging as the longer calculation times require the use of the parallel implementation of CrystalEstimator making direct comparison challenging. Nevertheless, the cost of the algorithm should scale almost linearly with the cost of the lattice minimizations. For the multipole models, approximately 1.85 s is required per lattice energy minimization, hence one can expect about an order of magnitude increase in computation cost per Sobol’ point optimization (Table S3).

## 6 Conclusions

In this work we have developed a framework for performing parameter estimation for a popular form of model commonly used in CSP applications. One of the main features of the methodology is the introduction of analytical relations for the derivatives of the parameter estimation merit function, which brings significant advantages over the numerical approaches commonly employed. The approach has required the development of new tools for the calculation of the intermolecular energy and for lattice minimizations, captured in the newly developed code CSO-RM.

The implementation of CSO-RM supports the use of both point-charge and multipole based models of the electrostatic interactions, allowing both types of models to be parametrized with our framework. We have shown elsewhere^11^ that the implementation of CSO-RM is remarkably efficient and robust, leading to superior performance over other programs of its kind, as well as providing greater accuracy in intermolecular energy calculations and finer control over the lattice minimization procedure. The implementation of CSO-RM as a software library also allows for easy interfacing of the code with other programs. This has been demonstrated in our implementation of the code CrystalEstimator for solving large-scale problems in parameter estimation using derivative based optimization techniques. A number of novel optimization techniques are also applied in CrystalEstimator to further ensure speed and robustness in the approach.

Reference crystal structure energies and geometries have been obtained from accurate DFT-D computations for a variety of structures containing the elements carbon, hydrogen, oxygen, nitrogen, fluorine, chlorine, and sulfur. In total, a database of 755 structures has been constructed, which can be reused to fit different models. Parameter estimates consistent with different electrostatic models or different forms of the empirical potential can therefore be obtained easily with our approach. This database has been made available for public use through Zenodo (doi: 10.5281/zenodo.7813566) making it one of the largest published databases of high-quality DFT-D resolved organic crystal structures. We have applied our approach to a number of test problems, demonstrating that problems containing up to 30 parameters and 140 structures in the training set can be solved readily to optimality. Larger problems become more challenging to solve due to discontinuities present in the parameter estimation merit function. Nevertheless it seems that the parameter estimation algorithm is successful in bringing the objective function close to the optimal solution of parameter estimation problem even when an optimal result cannot be obtained. Finally, analysis of the combined implementation of CSO-RM and CrystalEstimator has shown that the approach is highly efficient. The combined cost reductions from improved robustness, and more efficient and accurate gradient calculations reduces the computational cost of the entire methodology by approximately 3–4 orders of magnitude compared to alternative approaches. The walltime taken by the calculations is further reduced through parallelization of the code which can speed up the calculations by a further order of magnitude.

With the development of the tools outlined in this work, large-scale parameter estimation problems for the development of lattice energy force-fields can now be solved quickly and reliably for the first time. In combination with our comprehensive DFT-D reference dataset, this will allow us to not only develop new parameter estimates for CSP models, but also to test rigorously other factors in the model that influence model performance, such as the use of combining rules and different models of electrostatic energy. Using the database of reference crystal structures, this will be the focus of part 2 of this paper series.

## Data Availability

Data underlying this article can be accessed on Zenodo and used under the Creative Commons Attribution license. The CE-755 dataset may be found with doi: 10.5281/zenodo.7813566, and raw data related to the test problem performance with doi: 10.5281/zenodo.7836455.

## Supporting Information Available

Raw data for parameter estimation algorithm performance (PDF)
