# Supplementary material for: Large-Scale Parameter Estimation for Crystal Structure Prediction. Part 1: Dataset, Methodology, and Implementation
Source: J Chem Theory Comput. 2024 Nov 12;20(22):10288–315. doi: 10.1021/acs.jctc.4c01091 (PMC11603618; doi:10.1021/acs.jctc.4c01091)
Supplement: Supplementary file 1 — ct4c01091_si_001.pdf [file ct4c01091_si_001.pdf]

# Electronic Supporting Information

## Large-scale parameter estimation for Crystal Structure Prediction. Part 1: Dataset, Methodology, and Implementation

D. H. Bowskill[1†], B. I. Tan[1], A. Keates[2], I. J. Sugden[1‡], C. S. Adjiman[1], and C. C. Pantelides[1*]

[1] *Department of Chemical Engineering, Sargent Centre for Process Systems Engineering and Institute for Molecular Science and Engineering, Imperial College London, London SW7 2AZ, United Kingdom*

[2] *Process Studies Group, Syngenta, Jealott's Hill International Research Centre, Bracknell, Berkshire, RG42 6EY, United Kingdom*

October 29, 2024

# Contents

The CE755 Crystal Structure Database 2

Selection of lattice energy minimisation settings 2

*Ab initio* calculations 2

Selection of parameter estimation numerical settings 3

Intermolecular hydrogen bond analysis 3

Comparison of experimental and DFT-D lattice energies 3

Optimisation performance of multipole model parameter estimation 7

**Present Addresses:**

†Syngenta Crop Protection, Breitenloh 5, Münchwilen CH-4333, Switzerland

‡The Cambridge Crystallographic Data Centre, 12 Union Road, Cambridge, UK

***Corresponding Author:** c.pantelides@imperial.ac.uk

# The CE755 Crystal Structure Database

The compiled list of all structures included in the CE755 reference dataset can be found in the corresponding Crystal_Structure_Database.xlsx file. This includes basic information regarding the optimised crystal energies and geometries. The optimised crystal structures, in the form of .res files, can be accessed through Zenodo (doi: 10.5281/zenodo.7813566).

# Selection of intermolecular energy minimisation settings

The intermolecular energies calculated by CSO-RM require the evaluation of both the repulsion/dispersion contribution and the electrostatic contribution to intermolecular energy. For the repulsion/dispersion summations, only real-space summations need to be considered as there are no complications related to conditional convergence. A 15 Å cutoff limit is imposed on these interactions. On the other hand, electrostatic interactions can exhibit conditional convergence. Specifically, electrostatic interactions between multipoles with combined rank $l_1 + l_2 \leq 2$ are conditionally convergent while higher-order multipole interactions are not. To accurately evaluate these contributions, a generalised Ewald summation is employed [1, 2] on *all* ranks of multipole interactions, with a limit of $l_1 + l_2 \leq 4$. Within the Ewald summation, the real space summation for $l_1 + l_2 = 0$ interactions is set at 10 Å while the higher multipole summation limits and the reciprocal space summation limits are determined following the methodology of Bowskill [3] with a minimum acceptable accuracy of $\epsilon = 10^{-6}$ kJ/mol. Finally, a quintic spline [3] is applied to the last 10% of each of the summations (repulsion/dispersion and electrostatic).

For a set of repulsion/dispersion parameters ($\boldsymbol{p}$), the parameter estimation algorithm determines the objective function value and minimisation trajectory using the optimised geometry and energies of the crystal structures used for training ($\boldsymbol{\Omega}_s^*$ and $U_{inter,s}^*$ respectively). In order to diminish the problem of discontinuities in the parameter estimation objective function surface, these values must be determined to high precision. To that end, an extremely tight tolerance is used with the modified-Newton optimisation algorithm implemented in CSO-RM [3] such that convergence is reached when the norm of the gradient vector of the intermolecular energy is less than $10^{-10}$. In principle, this Newton algorithm could be used throughout the energy minimisation of a crystal structure, but this would add to the computational costs. Instead, the Newton algorithm is preceded by the NAG E04UFF algorithm, which is a quasi-Newton minimisation routine [4]. A optimality tolerance of $10^{-12}$ is used in this subroutine in order to bring the energy minimisation as close as possible to the true solution, reducing the number of Newton algorithm iterations required to reach convergence.

# *Ab initio* calculations

For the determination of $U_{elec}$ contributions, it is necessary to obtain a description of the point-charge and/or multipoles centered on each atomic site. Charge density calculations are performed at the PBE0 6-311G(d,p) level of theory in Gaussian 09 [5]. All self-consistent field calculations are conducted with Gaussian's 'Ultrafine' integration grid and 'YQC' algorithm. By default, Gaussian will attempt to leverage molecular symmetry in its calculations. This has been disabled using the 'Symmetry=None' keywords.

Values for atom-centred point charges are derived from the charge density calculations using the HLYGAt fitting scheme [6] implemented in Gaussian 09. Equivalently, atom-centred multipoles are derived from the same SCF calculations using distributed multipole analysis [7, 8] as implemented in

GDMA2.2. Within GDMA, 'SWITCH 4' is used in order to utilise the grid-based quadrature distribution method [8]. 'RADIUS H 0.65' is also applied for all GDMA calculations. In the HAIEFF formulation, it is assumed that these electrostatic features are independent of the crystalline environment and are only affected by the molecular conformation. Because only rigid minimisations are conducted during the parameter estimation, the derivation of the electrostatic features (point-charge or multipole) of all structures is done in advance of the parameter estimation and these features are fixed throughout the parameter estimation.

## Selection of parameter estimation numerical settings

For the parameter estimation, multistart optimisation is applied with Sobol' sampling [9] used to generate initial parameter vectors, $\bar{\boldsymbol{p}}^{[0]}$. The parameter space within which initial parameter vectors are generated is defined with respect to a reference set of parameter values. In this work, this reference is selected as the FIT potential values, where the parameters for Hn interactions are used for the hydrogen-polarised (Hp) environment, and the sulphur parameters are those used by the Price group in the first crystal structure prediction blind test [10]. The sampling is conducted with a region of ±40% of the reference values. The coverage achieved by Sobol' sampling is most effective when 2 to the power of an integer number of samples are performed. As a standard approach, parameters are generated using the first $2^7 = 128$ Sobol' points to sample the space of parameter estimates. Because the first Sobol' seed generated corresponds to the 'centre' of the sampling space, the first Sobol' seed is always identical to the reference parameter values. For each optimisation, a tight convergence tolerance of $10^{-8}$ is selected for the E04USA algorithm [4]. Up to 5 reinitialisations of the algorithm and 20 structure removals are permitted for each Sobol' point optimisation.

## Intermolecular hydrogen bond analysis

The CCDC's Python API is used to analyse the DFT-D optimised structures. The 'hbonds' crystal property generates a list of unique crystallographic intermolecular hydrogen bonds based on several geometric criteria. In our analysis, we keep all the default values for the geometric criteria except that we expand the lower bound of the angle tolerance from 120° to 100°. The use of these geometric criteria to define and identify hydrogen bonds is a commonly accepted approach and it is generally considered the most practical way to assess hydrogen bond formation [11].

## Comparison of experimental and DFT-D lattice energies

As described in Section 5 of the main text, accuracy of the DFT-D calculated lattice energies ($U_{latt}^{DFT}$) was benchmarked against experimentally-determined lattice energies ($U_{latt}^{exp}$). To obtain the latter, experimentally-determined sublimation energies ($\Delta H_{sub}^{exp}$) measured at finite-temperature, $T$, are corrected to static, 0 K lattice energies using the Dulong-Petit law ($U_{latt}^{exp} \approx -\Delta H_{sub}^{exp} - 2RT$) [12, 13]. All the experimental data used in this benchmarking are tabulated in Table S1 together with their corresponding DFT-D energies.

Table S1: Comparison of experimentally-derived lattice energies ($U_{latt}^{exp} \approx -\Delta H_{sub}^{exp} - 2RT$) with the DFT-D computed lattice energies ($U_{latt}^{DFT}$). The errors are computed as ($\Delta U_{latt} = U_{latt}^{exp} - U_{latt}^{DFT}$). In some cases, the same structure is referenced using different REFCODEs in our database (CE755) and in the experimental data.

| CE755 REFCODE | $U_{latt}^{DFT}$ (kJ/mol) | Exp. REFCODE | Source | $\Delta H_{sub}^{exp}$ (kJ/mol) | $T$ (K) | $U_{latt}^{exp}$ (kJ/mol) | $\Delta U_{latt}$ (kJ/mol) |
|---|---|---|---|---|---|---|---|
| ACETAC07 | -73.15 | ACETAC07 | [14] | 67.00 | 221.50 | -70.68 | 2.46 |
| ADAMAN08 | -69.28 | ADAMAN08 | [15] | 59.00 | 298.15 | -63.96 | 5.32 |
| AMBNAC12 | -125.91 | AMBNAC04 | [16] | 116.10 | 298.15 | -121.06 | 4.85 |
| AMBNAC12 | -125.91 | AMBNAC04 | [17] | 116.10 | 298.15 | -121.06 | 4.85 |
| AMPYRE | -104.43 | AMPYRE | [18] | 87.10 | 298.15 | -92.06 | 12.37 |
| AMPYRE | -104.43 | AMPYRE01 | [19] | 88.10 | 298.15 | -93.06 | 11.37 |
| AMPYRE | -104.43 | AMPYRE01 | [18] | 87.10 | 298.15 | -92.06 | 12.37 |
| AMPYRM11 | -96.08 | AMPYRM11 | [18] | 76.50 | 298.15 | -81.46 | 14.62 |
| ANTCEN16 | -113.00 | ANTCEN16 | [20] | 97.90 | 298.15 | -102.86 | 10.14 |
| BAGFIY01 | -102.83 | BAGFIY01 | [21] | 85.50 | 298.15 | -90.46 | 12.37 |
| BENZAC12 | -102.67 | BENZAC12 | [22] | 90.00 | 298.15 | -94.96 | 7.71 |
| BENZEN06 | -55.02 | BENZEN06 | [23] | 44.40 | 298.15 | -49.36 | 5.66 |
| BESKAL04 | -127.39 | BESKAL04 | [24] | 130.40 | 298.15 | -135.36 | -7.96 |
| BIPYRL04 | -96.88 | BIPYRL04 | [25] | 81.80 | 298.15 | -86.76 | 10.13 |
| BIPYRL04 | -96.88 | BIPYRL04 | [26] | 81.90 | 298.15 | -86.86 | 10.03 |
| BIPYRL04 | -96.88 | BIPYRL04 | [27] | 75.00 | 298.15 | -79.96 | 16.93 |
| BNZQUI03 | -76.24 | BNZQUI03 | [28] | 66.70 | 298.15 | -71.66 | 4.58 |
| BNZQUI03 | -76.24 | BNZQUI03 | [28] | 66.70 | 298.15 | -71.66 | 4.58 |
| BNZQUI03 | -76.24 | BNZQUI03 | [29] | 62.80 | 298.15 | -67.76 | 8.48 |
| BNZQUI03 | -76.24 | BNZQUI03 | [30] | 62.76 | 298.15 | -67.72 | 8.52 |
| BZDMAZ02 | -111.69 | BZDMAZ02 | [31] | 101.20 | 298.15 | -106.16 | 5.53 |
| BZDMAZ02 | -111.69 | BZDMAZ02 | [32] | 107.00 | 298.15 | -111.96 | -0.27 |
| BZDMAZ02 | -111.69 | BZDMAZ02 | [31] | 102.20 | 298.15 | -107.16 | 4.53 |
| BZDMAZ02 | -111.69 | BZDMAZ02 | [33] | 98.90 | 298.15 | -103.86 | 7.83 |
| BZDMAZ02 | -111.69 | BZDMAZ02 | [34] | 94.30 | 298.15 | -99.26 | 12.43 |
| BZPHAN01 | -124.38 | BZPHAN01 | [35] | 106.00 | 298.15 | -110.96 | 13.42 |
| CLBZAC01 | -116.31 | CLBZAC01 | [36] | 106.30 | 298.15 | -111.26 | 5.05 |
| CLBZAC01 | -116.31 | CLBZAC01 | [37] | 100.90 | 298.15 | -105.86 | 10.45 |
| CLMETH03 | -33.15 | CLMETH03 | [38] | 31.60 | 151.00 | -34.11 | -0.96 |
| CRYSEN | -138.77 | CRYSEN | [39] | 123.40 | 298.15 | -128.36 | 10.41 |
| CUBANE | -56.48 | CUBANE | [40] | 55.20 | 298.15 | -60.16 | -3.68 |
| CUKCIU03 | -54.38 | CUKCIU03 | [41] | 35.60 | 272.00 | -40.12 | 14.26 |
| CYHEXO | -86.79 | CYHEXO | [42] | 75.00 | 298.15 | -79.96 | 6.83 |
| DCLANT10 | -123.74 | DCLANT10 | [43] | 113.90 | 346.00 | -119.65 | 4.09 |
| DCLBQN | -82.73 | DCLBQN | [30] | 69.90 | 298.15 | -74.86 | 7.88 |
| DITHAN02 | -84.60 | DITHAN02 | [44] | 63.00 | 298.15 | -67.96 | 16.65 |
| DITHAN02 | -84.60 | DITHAN01 | [33] | 68.90 | 298.15 | -73.86 | 10.75 |

Continued on next page

Table S1 – continued from previous page

| CE755 REFCODE | $U_{latt}^{DFT}$ (kJ/mol) | Exp. REFCODE | Source | $\Delta H_{sub}^{exp}$ (kJ/mol) | $T$ (K) | $U_{latt}^{exp}$ (kJ/mol) | $\Delta U_{latt}$ (kJ/mol) |
|---|---|---|---|---|---|---|---|
| DITHAN02 | -84.60 | DITHAN01 | [44] | 63.00 | 298.15 | -67.96 | 16.65 |
| DMSULO04 | -83.79 | DMSULO02 | [45] | 77.00 | 298.15 | -81.96 | 1.83 |
| DNBENZ11 | -85.32 | DNBENZ11 | [46] | 87.00 | 298.15 | -91.96 | -6.63 |
| DTENYL02 | -96.43 | DTENYL02 | [47] | 85.20 | 298.15 | -90.16 | 6.27 |
| FABPON11 | -138.61 | FABPON11 | [48] | 123.30 | 298.15 | -128.26 | 10.35 |
| FORMAC02 | -67.58 | FORMAC02 | [15] | 60.50 | 274.50 | -65.06 | 2.51 |
| FULPIM | -96.27 | FULPIM | [49] | 88.40 | 298.15 | -93.36 | 2.91 |
| HCLBNZ13 | -100.14 | HCLBNZ13 | [43] | 90.50 | 298.15 | -95.46 | 4.69 |
| HEXANE01 | -51.67 | HEXANE01 | [50] | 50.80 | 178.00 | -53.76 | -2.09 |
| HXMTAM10 | -85.17 | HXMTAM10 | [51] | 79.60 | 298.15 | -84.56 | 0.61 |
| HXMTAM10 | -85.17 | HXMTAM08 | [52] | 74.90 | 298.15 | -79.86 | 5.31 |
| HXQUIN14 | -91.43 | HXQUIN11 | [53] | 109.00 | 298.15 | -113.96 | -22.52 |
| HXQUIN14 | -91.43 | HXQUIN11 | [54] | 108.80 | 298.15 | -113.76 | -22.32 |
| HXQUIN14 | -91.43 | HXQUIN11 | [55] | 89.50 | 298.15 | -94.46 | -3.02 |
| HXQUIN14 | -91.43 | HXQUIN11 | [55] | 89.00 | 298.15 | -93.96 | -2.52 |
| HXQUIN14 | -91.43 | HXQUIN11 | [53] | 87.90 | 298.15 | -92.86 | -1.42 |
| IMAZOL06 | -91.12 | IMAZOL06 | [31] | 81.40 | 298.15 | -86.36 | 4.76 |
| MALIAC12 | -117.02 | MALIAC12 | [56] | 105.40 | 298.15 | -110.36 | 6.66 |
| MELAMI06 | -150.94 | MELAMI06 | [15] | 123.30 | 432.00 | -130.48 | 20.46 |
| MELAMI06 | -150.94 | MELAMI05 | [57] | 120.00 | 298.15 | -124.96 | 25.98 |
| NAPHTA31 | -83.65 | NAPHTA31 | [58] | 72.70 | 298.15 | -77.66 | 5.99 |
| NAPHTA31 | -83.65 | NAPHTA15 | [59] | 73.20 | 298.15 | -78.16 | 5.49 |
| NAPHTA31 | -83.65 | NAPHTA15 | [60] | 73.00 | 298.15 | -77.96 | 5.69 |
| NAPHTA31 | -83.65 | NAPHTA15 | [61] | 72.90 | 298.15 | -77.86 | 5.79 |
| NAPHTA31 | -83.65 | NAPHTA15 | [62] | 72.70 | 298.15 | -77.66 | 5.99 |
| NAPHTA31 | -83.65 | NAPHTA15 | [39] | 72.60 | 298.15 | -77.56 | 6.09 |
| NAPHTA31 | -83.65 | NAPHTA15 | [63] | 72.60 | 298.15 | -77.56 | 6.09 |
| NAPHTA31 | -83.65 | NAPHTA15 | [64] | 72.50 | 298.15 | -77.46 | 6.19 |
| NAPHTA31 | -83.65 | NAPHTA15 | [65] | 72.40 | 298.15 | -77.36 | 6.29 |
| NAPHTA31 | -83.65 | NAPHTA15 | [66] | 72.30 | 298.15 | -77.26 | 6.39 |
| NAPHTA31 | -83.65 | NAPHTA15 | [67] | 72.10 | 298.15 | -77.06 | 6.59 |
| NAPHTA31 | -83.65 | NAPHTA15 | [68] | 70.40 | 298.15 | -75.36 | 8.29 |
| NAPHTA31 | -83.65 | NAPHTA15 | [69] | 66.50 | 298.15 | -71.46 | 12.19 |
| NAPHTA31 | -83.65 | NAPHTA15 | [33] | 64.00 | 298.15 | -68.96 | 14.69 |
| NAPOAC01 | -127.98 | NAPOAC01 | [16] | 113.60 | 298.15 | -118.56 | 9.42 |
| OCTANE10 | -67.34 | OCTANE10 | [50] | 68.10 | 216.00 | -71.69 | -4.35 |
| PENTAN01 | -43.33 | PENTAN01 | [50] | 42.00 | 143.00 | -44.38 | -1.05 |
| PERLEN06 | -148.57 | PERLEN06 | [70] | 129.60 | 415.00 | -136.50 | 12.07 |
| PFBZAC | -92.12 | PFBZAC01 | [71] | 91.50 | 298.15 | -96.46 | -4.34 |
| PHENAN08 | -106.09 | PHENAN08 | [66] | 90.90 | 298.15 | -95.86 | 10.23 |
| PHENAZ04 | -106.40 | PHENAZ04 | [72] | 94.30 | 354.00 | -100.19 | 6.22 |

Continued on next page

Table S1 – continued from previous page

| CE755 REFCODE | $U_{latt}^{DFT}$ (kJ/mol) | Exp. REFCODE | Source | $\Delta H_{sub}^{exp}$ (kJ/mol) | $T$ (K) | $U_{latt}^{exp}$ (kJ/mol) | $\Delta U_{latt}$ (kJ/mol) |
|---|---|---|---|---|---|---|---|
| PNCLBZ | -93.14 | PNCLBZ | [73] | 87.03 | 298.00 | -91.98 | 1.16 |
| PRMDIN01 | -65.09 | PRMDIN02 | [73] | 58.30 | 298.15 | -63.26 | 1.83 |
| PTOXEC | -76.73 | PTOXEC | [74] | 87.90 | 298.15 | -92.86 | -16.12 |
| PTOXEC | -76.73 | PTOXEC | [73] | 87.86 | 298.00 | -92.82 | -16.09 |
| PYRAZI01 | -65.26 | PYRAZI01 | [75] | 56.20 | 298.15 | -61.16 | 4.10 |
| PYRENE03 | -113.74 | PYRENE03 | [39] | 100.30 | 298.15 | -105.26 | 8.49 |
| SALMID04 | -115.45 | SALMID02 | [76] | 101.90 | 298.15 | -106.86 | 8.59 |
| SALMID04 | -115.45 | SALMID02 | [77] | 99.30 | 298.15 | -104.26 | 11.19 |
| SUCACB03 | -132.44 | SUCACB03 | [78] | 123.10 | 298.15 | -128.06 | 4.38 |
| SUCANH12 | -78.54 | SUCANH12 | [79] | 80.70 | 298.15 | -85.66 | -7.12 |
| SUCANH12 | -78.54 | SUCANH12 | [79] | 80.70 | 298.15 | -85.66 | -7.12 |
| TCYETY01 | -85.42 | TCYETY01 | [80] | 81.20 | 298.15 | -86.16 | -0.73 |
| TETDAM03 | -70.45 | TETDAM03 | [81] | 62.20 | 298.15 | -67.16 | 3.29 |
| TOXOCN | -72.01 | TOXOCN | [82] | 79.60 | 298.15 | -84.56 | -12.55 |
| TOXOCN | -72.01 | TOXOCN | [73] | 79.50 | 298.00 | -84.45 | -12.44 |
| TRAZOL03 | -91.28 | TRAZOL03 | [83] | 83.00 | 298.15 | -87.96 | 3.32 |
| TRAZOL03 | -91.28 | TRAZOL03 | [73] | 84.10 | 298.00 | -89.05 | 2.22 |
| TRIPHE13 | -133.93 | TRIPHE13 | [39] | 120.10 | 298.15 | -125.06 | 8.88 |
| TRITAN03 | -109.71 | TRITAN03 | [84] | 93.20 | 298.15 | -98.16 | 11.56 |
| TRIZIN01 | -59.88 | TRIZIN01 | [85] | 54.20 | 298.15 | -59.16 | 0.72 |
| TRIZIN01 | -59.88 | TRIZIN01 | [73] | 58.16 | 222.00 | -61.85 | -1.97 |
| TRIZIN01 | -59.88 | TRIZIN01 | [33] | 56.50 | 298.15 | -61.46 | -1.58 |
| TRIZIN01 | -59.88 | TRIZIN01 | [85] | 54.18 | 298.15 | -59.14 | 0.74 |
| TROXAN | -56.98 | TROXAN | [86] | 56.60 | 298.15 | -61.56 | -4.58 |
| XAGWIM | -112.43 | XAGWIM | [87] | 100.80 | 298.15 | -105.76 | 6.67 |
| ZZZKPE03 | -103.02 | ZZZKPE03 | [88] | 85.90 | 343.00 | -91.60 | 11.42 |

# Optimisation performance of multipole model parameter estimation

The performance tests conducted on the point-charge based electrostatic models is repeated for models using multipole electrostatics. The same seven tests problems are first investigated in terms of their convergence behaviour (Table S2). The convergence behaviour is classified according to the criteria described in Figure 1 of the main text. For the most part, the convergence behaviour is similar to their point-charge counterparts.

Table S2: Overview of optimisation results for all seven test problems from 128 separate Sobol' points for multipole-based models. The termination criteria are as described in Figure 1 of the main text.

| Test Problem | Runs terminated by criteria B3 | Runs terminated by criteria B4 or B5 | Failed optimisations | Unique minima |
|---|---|---|---|---|
| TP1 | 120 | 8 | 0 | 2 |
| TP2 | 121 | 7 | 0 | 1 |
| TP3 | 120 | 8 | 0 | 1 |
| TP4 | 117 | 10 | 1 | 3 |
| TP5 | 102 | 24 | 2 | 1 |
| TP6 | 11 | 117 | 0 | 2 |
| TP7 | 10 | 114 | 4 | 3 |

Following this, we also consider the computational cost (Table S3) of conducting these parameter estimations. Unlike the parameter estimation for the point-charge based models, which could be conducted serially, all of the parameter estimation conducted for the multipole based models were done with the parallel implementation of CrystalEstimator. The final walltime reported by CrystalEstimator reflects the time from which the parameter estimation starts till when it ends. This does not distinguish between time where the workers are actively carrying out lattice energy minimisations and time where workers may be idle. Unfortunately, there is no convenient alternative that can be used to more accurately quantify computational cost. Using this reported time, it is evident that the parameter estimation takes a significantly greater amount of time to complete for the multipole models, in large part due to the added computational complexity of the multipole calculations. Fitting a linear correlation between the average number of lattice minimisations and the average walltime, the cost per lattice energy minimisation is about 1.85 seconds. This is almost four times more expensive than for the point-charge models.

Table S3: Overview of performance statistics for all seven test problems using the modified local optimisation procedure from 128 separate Sobol' points for multipole-based models.

| Test Problem | Average no. of total merit function evaluations | Average no. of lattice minimisations | Average walltime (hr:min:sec) |
|---|---|---|---|
| TP1 | 36.8 | 663.1 | 00:32:09 |
| TP2 | 23.2 | 1,113.6 | 01:29:32 |
| TP3 | 21.7 | 2,083.2 | 01:43:52 |
| TP4 | 37.9 | 5,311.6 | 03:24:17 |
| TP5 | 40.1 | 9,189.8 | 04:33:04 |
| TP6 | 43.7 | 13,668.7 | 08:25:35 |
| TP7 | 52.1 | 23,193.4 | 10:53:49 |

# References

[1] Nijboer, B. R. A.; De Wette, F. W. On the calculation of lattice sums. *Physica* **1957**, *23*, 309–321.

[2] Vasileiadis, M. Calculation of the free energy of crystalline solids. Ph.D. thesis, Imperial College London London, UK, 2013.

[3] Bowskill, D. H. Reliable and Efficient Parameter Estimation Methodologies for Crystal Structure Prediction. Ph.D. thesis, Imperial College London, 2021.

[4] The Numerical Algorithms Group (NAG) The NAG Library for Fortran. `https://www.nag.com`, Last accessed on 16.03.2021.

[5] Frisch, M. J. et al. Gaussian 09, Revision C.01. 2010.

[6] Hu, H.; Lu, Z.; Yang, W. Fitting molecular electrostatic potentials from quantum mechanical calculations. *Journal of Chemical Theory and Computation* **2007**, *3*, 1004–1013.

[7] Stone, A. J. Distributed multipole analysis, or how to describe a molecular charge distribution. *Chemical Physics Letters* **1981**, *83*, 233–239.

[8] Stone, A. J. Distributed multipole analysis: Stability for large basis sets. *Journal of Chemical Theory and Computation* **2005**, *1*, 1128–1132.

[9] Sobol', I. M. On the distribution of points in a cube and the approximate evaluation of integrals. *Zhurnal Vychislitel'noi Matematiki i Matematicheskoi Fiziki* **1967**, *7*, 784–802.

[10] Lommerse, J. P. M.; Motherwell, W. D. S.; Ammon, H. L.; Dunitz, J. D.; Gavezzotti, A.; Hofmann, D. W. M.; Leusen, F. J. J.; Mooij, W. T. M.; Price, S. L.; Schweizer, B.; Schmidt, M. U.; van Eijck, B. P.; Verwer, P.; Williams, D. E. A test of crystal structure prediction of small organic molecules. *Acta Crystallographica Section B: Structural Science* **2000**, *56*, 697–714.

[11] Bernstein, J.; Davis, R. E.; Shimoni, L.; Chang, N.-L. Patterns in Hydrogen Bonding: Functionality and Graph Set Analysis in Crystals. *Angewandte Chemie International Edition in English* **1995**, *34*, 1555–1573.

[12] Gatsiou, C.-A. Improving the accuracy of lattice energy calculations in crystal structure prediction using experimental data. Ph.D. thesis, Imperial College London, 2016.

[13] Reilly, A. M.; Tkatchenko, A. Understanding the role of vibrations, exact exchange, and many-body van der Waals interactions in the cohesive properties of molecular crystals. *The Journal of Chemical Physics* **2013**, *139*, 024705.

[14] Calis-Van Ginkel, C. H. D.; Calis, G. H. M.; Timmermans, C. W. M.; de Kruif, C. G.; Oonk, H. A. J. Enthalpies of sublimation and dimerization in the vapour phase of formic, acetic, propanoic, and butanoic acids. *The Journal of Chemical Thermodynamics* **1978**, *10*, 1083–1088.

[15] Stephenson, R. M.; Malanowski, S. K. *Handbook of the thermodynamics of organic compounds*; Springer, 1987.

[16] Sabbah, R.; Chastel, R.; Laffitte, M. Thermodynamique de composés azotés. I. Etude calorimétrique des enthalpies de sublimation des trois acides aminobenzoïques. *Canadian Journal of Chemistry* **1974**, *52*, 2201–2205.

[17] Nabavian, M.; Sabbah, R.; Chastel, R.; Laffitte, M. Thermodynamique de composés azotés - II. - Étude thermochimique des acides aminobenzoïques, de la pyrimidine, de l'uracile et de la thymine. *J. Chim. Phys.* **1977**, *74*, 115–126.

[18] Sabbah, R.; da Silva Eusébio, M. E. Energétique des liaisons inter- et intramoléculaires dans les trois isomères de l'aminopyridine. *Canadian Journal of Chemistry* **1998**, *76*, 18–24.

[19] Bickerton, J.; Pilcher, G.; Al-Takhin, G. Enthalpies of combustion of the three aminopyridines and the three cyanopyridines. *The Journal of Chemical Thermodynamics* **1984**, *16*, 373–378.

[20] Oja, V.; Chen, X.; Hajaligol, M. R.; Chan, W. G. Sublimation Thermodynamic Parameters for Cholesterol, Ergosterol, $\beta$-Sitosterol, and Stigmasterol. *Journal of Chemical & Engineering Data* **2009**, *54*, 730–734.

[21] Sabbah, R.; Perez, L. Energétique des liaisons inter- et intramoléculaires dans les trois isomères du benzènediamine. *Canadian Journal of Chemistry* **1997**, *75*, 357–364.

[22] Linstrom, P. J.; Mallard, W. G. The NIST Chemistry WebBook: A Chemical Data Resource on the Internet. *Journal of Chemical & Engineering Data* **2001**, *46*, 1059–1063.

[23] De Kruif, C. G. Enthalpies of sublimation and vapour pressures of 11 polycyclic hydrocarbons. *The Journal of Chemical Thermodynamics* **1980**, *12*, 243–248.

[24] Monte, M. J. S.; Gonçalves, M. V.; Ribeiro da Silva, M. D. M. C. Vapor Pressures and Enthalpies of Combustion of the Dihydroxybenzoic Acid Isomers. *Journal of Chemical & Engineering Data* **2010**, *55*, 2246–2251.

[25] Ribeiro da Silva, M. A. V.; Morais, V. M. F.; Matos, M. A. R.; Rio, C. M. A. Thermochemical and Theoretical Studies of Some Bipyridines. *The Journal of Organic Chemistry* **1995**, *60*, 5291–5294.

[26] Faour, M.; Akasheh, T. S. Heat of combustion of some N-heterocycle compounds. Part 1. *J. Chem. Soc., Perkin Trans. 2* **1985**, 811–813.

[27] Emmenegger, F. The Complex of Co(2,2,6,6-tetramethyl-3,5-heptanedionate)2 with 2,2'-Bipyridine. Its Formation in the Gas Phase and in Solution. *Inorganic Chemistry* **1996**, *35*, 5931–5934.

[28] Rojas-Aguilar, A.; Flores-Lara, H.; Martinez-Herrera, M.; Ginez-Carbajal, F. Thermochemistry of benzoquinones. *The Journal of Chemical Thermodynamics* **2004**, *36*, 453–463.

[29] Magnus, A. Die Resonanzenergien der Parachinone. *Zeitschrift für Physikalische Chemie* **1956**, *9*, 141–161.

[30] Coolidge, A. S.; Coolidge, M. S. The Sublimation Pressures of Substituted Quinones and Hydroquinones. *Journal of the American Chemical Society* **1927**, *49*, 100–104.

[31] Jiménez, P.; Roux, M. V.; Turrión, C.; Gomis, F. Thermochemical properties of N-heterocyclic compounds I. Enthalpies of combustion, vapour pressures and enthalpies of sublimation, and enthalpies of formation of pyrazole, imidazole, indazole, and benzimidazole. *The Journal of Chemical Thermodynamics* **1987**, *19*, 985–992.

[32] Zimmermann, H.; Geisenfelder, H. Über die Mesomerieenergie von Azolen. *Zeitschrift für Elektrochemie, Berichte der Bunsengesellschaft für physikalische Chemie* **1961**, *65*, 368–371.

[33] Acree, J., W.; Chickos, J. S. *NIST Chemistry WebBook, NIST Standard Reference Database Number 69*; National Institute of Standards and Technology: Gaithersburg MD, 2023.

[34] Lehman, R. L.; Gentry, J. S.; Glumac, N. G. Thermal stability of potassium carbonate near its melting point. *Thermochimica Acta* **1998**, *316*, 1–9.

[35] Wakayama, N.; Inokuchi, H. Heats of Sublimation of Polycyclic Aromatic Hydrocarbons and Their Molecular Packings. *Bulletin of the Chemical Society of Japan* **1967**, *40*, 2267–2271.

[36] Ribeiro da Silva, M. A. V.; Fonseca, J. M. S.; Carvalho, R. P. B. M.; Monte, M. J. S. Thermodynamic study of the sublimation of six halobenzoic acids. *The Journal of Chemical Thermodynamics* **2005**, *37*, 271–279.

[37] Sabbah, R.; Aguilar, A. R. Étude thermodynamique des trois isomères de l'acide chlorobenzoïque. Partie II. *Canadian Journal of Chemistry* **1995**, *73*, 1538–1545.

[38] Domalski, E. S.; Hearing, E. D. Heat Capacities and Entropies of Organic Compounds in the Condensed Phase. Volume III. *Journal of Physical and Chemical Reference Data* **1996**, *25*, 1–1.

[39] Roux, M. V.; Temprado, M.; Chickos, J. S.; Nagano, Y. Critically Evaluated Thermochemical Properties of Polycyclic Aromatic Hydrocarbons. *Journal of Physical and Chemical Reference Data - J Phys Chem Ref Data* **2008**, *37*, 1855–1996.

[40] Bashir-Hashemi, A.; Chickos, J. S.; Hanshaw, W.; Zhao, H.; Farivar, B. S.; Liebman, J. F. The enthalpy of sublimation of cubane. *Thermochimica Acta* **2004**, *424*, 91–97.

[41] Stull, D. R. Vapor Pressure of Pure Substances. Organic and Inorganic Compounds. *Industrial & Engineering Chemistry* **1947**, *39*, 517–540.

[42] Pilcher, G.; Parchment, O. G.; Hillier, I. H.; Heatley, F.; Fletcher, D.; Ribeiro da Silva, M. A. V.; Ferrao, M. L. C. C. H.; Monte, M. J. S.; Fang, J. Thermochemical and theoretical studies on cyclohexanediones. *The Journal of Physical Chemistry* **1993**, *97*, 243–247.

[43] Sabbah, R.; An, X. W. Etude thermodynamique des chlorobenzenes. *Thermochimica Acta* **1991**, *179*, 81–88.

[44] Dávalos, J. Z.; Flores, H.; Jiménez, P.; Notario, R.; Roux, M. V.; Juaristi, E.; Hosmane, R. S.; Liebman, J. F. Calorimetric, Computational (G2(MP2) and G3) and Conceptual Study of the Energetics of the Isomeric 1,3- and 1,4-Dithianes. *The Journal of Organic Chemistry* **1999**, *64*, 9328–9336.

[45] Busfield, W. K.; Mackle, H.; O'Hare, P. A. G. Studies in the thermochemistry of sulphones. Part 2. — The standard heats of formation of sulphones of the type RSO2CH3·. *Trans. Faraday Soc.* **1961**, *57*, 1054–1057.

[46] Ferro, D.; Piacente, V.; Gigli, R.; D'Ascenzo, G. Determination of the vapour pressures of o-, m-, and p-dinitrobenzene by the torsion-effusion method. *The Journal of Chemical Thermodynamics* **1976**, *8*, 1137–1143.

[47] Ribeiro da Silva, M. A. V.; Santos, A. F. L. O. M.; Gomes, J. R. B.; Roux, M. V.; Temprado, M.; Jiménez, P.; Notario, R. Thermochemistry of Bithiophenes and Thienyl Radicals. A Calorimetric and Computational Study. *The Journal of Physical Chemistry A* **2009**, *113*, 11042–11050.

[48] Geiseler, G.; Quitzsch, K.; Rauh, H.-J.; Schaffernicht, H.; Walther, H.-J. Bildungsenthalpien und Mesomerieenergien von $\pi$-Bindungssystemen. 1. Mitteilung: Bildungsenthalpien und Mesomerieenergien einiger mehrkerniger Aromaten und verschiedener Pseudoazulene. *Berichte der Bunsengesellschaft für physikalische Chemie* **1966**, *70*, 551–556.

[49] Jiménez, P.; Roux, M. V.; Turrión, C. Thermochemical properties of N-heterocyclic compounds IV. Enthalpies of combustion, vapour pressures and enthalpies of sublimation, and enthalpies of formation of 2-methylimidazole and 2-ethylimidazole. *The Journal of Chemical Thermodynamics* **1992**, *24*, 1145–1149.

[50] Bondi, A. Heat of Sublimation of Molecular Crystals: A Catalog of Molecular Structure Increments. *Journal of Chemical & Engineering Data* **1963**, *8*, 371–381.

[51] Verevkin, S. P. Relationships among strain energies of mono- and poly-cyclic cyclohexanoid molecules and strain of their component rings. *The Journal of Chemical Thermodynamics* **2002**, *34*, 263–275.

[52] Wada, T.; Kishida, E.; Tomiie, Y.; Suga, H.; Seki, S.; Nitta, I. Crystal Structure and Thermodynamical Investigations of Triethylenediamine, C6H12N2. *Bulletin of the Chemical Society of Japan* **1960**, *33*, 1317–1318.

[53] Horton, G. R.; Wendlandt, W. W. The heats of dissociation of the 8-quinolinol and substituted 8-quinolinol metal chelates of uranium (VI), thorium (IV) and scandium (III). *Journal of Inorganic and Nuclear Chemistry* **1963**, *25*, 241–245.

[54] Sakiyama, M.; Nakano, T.; Seki, S. Enthalpies of Combustion of Organic Compounds. I. 8-Quinolinol. *Bulletin of the Chemical Society of Japan* **1975**, *48*, 1705–1708.

[55] Ribeiro da Silva, M. A. V.; Monte, M. J. S.; Matos, M. A. R. Enthalpies of combustion, vapour pressures, and enthalpies of sublimation of 8-hydroxyquinoline, 5-nitro-8-hydroxyquinoline, and 2-methyl-8-hydroxyquinoline. *The Journal of Chemical Thermodynamics* **1989**, *21*, 159–166.

[56] Arshadi, M. R. Determination of heats of sublimation of organic compounds by a mass spectrometric–knudsen effusion method. *J. Chem. Soc., Faraday Trans. 1* **1974**, *70*, 1569–1571.

[57] Hirt, R. C.; Steger, J. E.; Simard, G. L. Vapor pressure of 2,4,6-triamino-s-triazine (melamine). *Journal of Polymer Science* **1960**, *43*, 319–323.

[58] Chirico, R. D.; Knipmeyer, S. E.; Nguyen, A.; Steele, W. V. The thermodynamic properties to the temperature 700 K of naphthalene and of 2,7-dimethylnaphthalene. *The Journal of Chemical Thermodynamics* **1993**, *25*, 1461–1494.

[59] Speros, D. M.; Rossini, F. D. Heats of combustion and formation of naphthalene, the two methylnaphthalenes, cis and trans- decahydronaphthalene, and related compounds. *The Journal of Physical Chemistry* **1960**, *64*, 1723–1727.

[60] Irving, R. J. The standard enthalpy of sublimation of naphthalene. *The Journal of Chemical Thermodynamics* **1972**, *4*, 793–794.

[61] Coleman, D. J.; Pilcher, G. Heats of combustion of biphenyl, bibenzyl, naphthalene, anthracene and phenanthrene. *Trans. Faraday Soc.* **1966**, *62*, 821–827.

[62] Miller, G. A. Vapor Pressure of Naphthalene. Thermodynamic Consistency with Proposed Frequency Assignments. *Journal of Chemical & Engineering Data* **1963**, *8*, 69–72.

[63] Van Ekeren, P. J.; Jacobs, M. H. G.; Offringa, J. C. A.; De Kruif, C. Vapour-pressure measurements on trans-diphenylethene and naphthalene using a spinning-rotor friction gauge. *The Journal of Chemical Thermodynamics* **1983**, *15*, 409–417.

[64] Sinke, G. C. A method for measurement of vapor pressures of organic compounds below 0.1 Torr Naphthalene as a reference substance. *The Journal of Chemical Thermodynamics* **1974**, *6*, 311–316.

[65] Murata, S.; Sakiyama, M.; Seki, S. Construction and testing of a sublimation calorimetric system using a Calvet microcalorimeter. *The Journal of Chemical Thermodynamics* **1982**, *14*, 707–721.

[66] Torres-Gómez, L. A.; Barreiro-Rodríguez, G.; Galarza-Mondragón, A. A new method for the measurement of enthalpies of sublimation using differential scanning calorimetry. *Thermochimica Acta* **1988**, *124*, 229–233.

[67] Morawetz, E. Enthalpies of vaporization for a number of aromatic compounds. *The Journal of Chemical Thermodynamics* **1972**, *4*, 455–460.

[68] Chickos, J.; Hesse, D.; Hosseini, S.; Nichols, G.; Webb, P. Sublimation enthalpies at 298.15K using correlation gas chromatography and differential scanning calorimetry measurements. *Thermochimica Acta* **1998**, *313*, 101–110.

[69] Wolf, K. L.; Weghofer, H. Über Sublimationswärmen. *Zeitschrift für Physikalische Chemie* **1938**, *39B*, 194–208.

[70] Inokuchi, H.; Shiba, S.; Handa, T.; Akamatu, H. Heats of Sublimation of Condensed Polynuclear Aromatic Hydrocarbons. *Bulletin of the Chemical Society of Japan* **1952**, *25*, 299–302.

[71] Cox, J. D.; Gundry, H. A.; Harrop, D.; Head, A. J. Thermodynamic properties of fluorine compounds 9. Enthalpies of formation of some compounds containing the pentafluorophenyl group. *The Journal of Chemical Thermodynamics* **1969**, *1*, 77–87.

[72] Chirico, R. D.; Kazakov, A. F.; Steele, W. V. Thermodynamic properties of three-ring aza-aromatics. 1. Experimental results for phenazine and acridine, and mutual validation of experiments and computational methods. *The Journal of Chemical Thermodynamics* **2010**, *42*, 571–580.

[73] Marchese Robinson, R. L.; Geatches, D.; Morris, C.; Mackenzie, R.; Maloney, A. G. P.; Roberts, K. J.; Moldovan, A.; Chow, E.; Pencheva, K.; Vatvani, D. R. M. Evaluation of Force-Field Calculations of Lattice Energies on a Large Public Dataset, Assessment of Pharmaceutical Relevance, and Comparison to Density Functional Theory. *Journal of Chemical Information and Modeling* **2019**, *59*, 4778–4792.

[74] Månsson, M. Non-bonded Oxygen-Oxygen Interactions in 2,4,10-Trioxa-adamantane and 1,3,5,7,9-Pentoxecane. *Acta Chemica Scandinavica* **1974**, *28b*, 895–899.

[75] Sakoguchi, A.; Ueoka, R.; Kato, Y.; Arai, Y. Vapor Pressure of Alkylpyridines and Alkylpyrazines. *Journal of Chemical Engineering* **1995**, *21*, 219–223.

[76] Bernardes, C. E. S.; Minas da Piedade, M. E. Energetics of the O–H Bond and of Intramolecular Hydrogen Bonding in HOC6H4C(O)Y (Y =, CH3, CH2CHCH2, CCH, CH2F, NH2, NHCH3, NO2, OH, OCH3, OCN, CN, F, Cl, SH, and SCH3) Compounds. *The Journal of Physical Chemistry A* **2008**, *112*, 10029–10039.

[77] Ribeiro da Silva, M. D. M. C.; Araújo, N. R. M. Thermochemical studies on salicylaldehyde and salicylamide. *The Journal of Chemical Thermodynamics* **2007**, *39*, 1372–1376, Honour issue Professor J. Simões Redinha on the occasion of his 80th birthday.

[78] De Wit, H. G. M.; Van Miltenburg, J. C.; De Kruif, C. G. Thermodynamic properties of molecular organic crystals containing nitrogen, oxygen, and sulphur 1. Vapour pressures and enthalpies of sublimation. *The Journal of Chemical Thermodynamics* **1983**, *15*, 651–663.

[79] Meng-Yan, Y.; Pilcher, G. Enthalpies of combustion of succinic anhydride, glutaric anhydride, and glutarimide. *The Journal of Chemical Thermodynamics* **1990**, *22*, 893–898.

[80] Boyd, R. H. Thermochemistry of Cyanocarbons. *The Journal of Chemical Physics* **1963**, *38*, 2529–2535.

[81] Westrum, E. F. J.; Rapport, N. J.; Andrews, J. T. S. Enthalpies of formation for globular molecules III. Succinonitrile and triethylenediamine. *Journal of the American Chemical Society* **1971**, *93*, 4363–4365.

[82] Månsson, M.; Nakase, Y.; Sunner, S. The Enthalpies of Formation of Trioxane and Tetroxane. *Acta Chemica Scandinavica* **1969**, *23*, 56–60.

[83] Jiménez, P.; Roux, M. V.; Turrión, C. Thermochemical properties of N-heterocyclic compounds II. Enthalpies of combustion, vapour pressures, enthalpies of sublimation, and enthalpies of formation of 1,2,4-triazole and benzotriazole. *The Journal of Chemical Thermodynamics* **1989**, *21*, 759–764.

[84] Roux, M. V.; Jiménez, P.; Dávalos, J. Z.; Notario, R.; Juaristi, E. Calorimetric and Computational Study of 1,3,5-Trithiane. *The Journal of Organic Chemistry* **2001**, *66*, 5343–5351.

[85] Byström, K. The stabilization energy of 1,3,5-triazine derived from measurements of the enthalpies of combustion and sublimation. *The Journal of Chemical Thermodynamics* **1982**, *14*, 865–870.

[86] Bogdanova, K. A.; Berlin, A. A.; Kompaniets, V. Z.; Rakova, G. V.; Miroshnichenko, Y. A.; Lebedev, Y. A.; Yenikolopyan, N. S. Enthalpies of the homogeneous polymerization of trioxane and tetroxane. *Polymer Science U.S.S.R.* **1975**, *17*, 759–766.

[87] Santos, A. F. L. O. M.; Ribeiro da Silva, M. A. V. Experimental and Computational Study on the Molecular Energetics of 2-Pyrrolecarboxylic Acid and 1-Methyl-2-pyrrolecarboxylic Acid. *The Journal of Physical Chemistry A* **2009**, *113*, 9741–9750.

[88] Maria, T. M. R.; Costa, F. S.; Leitão, M. L. P.; Redinha, J. S. A calorimetric study of phase transitions for some cyclohexanediols. *Thermochimica Acta* **1995**, *269-270*, 405–413, Recent Advances in Thermal Analysis and Calorimetry.
